# Synthesis of New Shogaol Analogues as NRF2 Activators and Evaluation of Their Anti-Inflammatory Activity, Modes of Action and Metabolic Stability

**DOI:** 10.3390/antiox12020475

**Published:** 2023-02-13

**Authors:** Kit-Kay Mak, Zhang Shiming, Raghavendra Sakirolla, Madhu Katyayani Balijepalli, Albena T. Dinkova-Kostova, Ola Epemolu, Zulkefeli Mohd, Mallikarjuna Rao Pichika

**Affiliations:** 1 Department of Pharmaceutical Chemistry, School of Pharmacy, International Medical University, 126, Jalan Jalil Perkasa 19, Bukit Jalil, Kuala Lumpur 57000, Selangor, Malaysia; 2 Centre of Excellence for Bioactive Molecules and Drug Delivery, Institute for Research, Development & Innovation, International Medical University, 126, Jalan Jalil Perkasa 19, Bukit Jalil, Kuala Lumpur 57000, Selangor, Malaysia; 3 School of Postgraduate Studies, International Medical University, 126, Jalan Jalil Perkasa 19, Bukit Jalil, Kuala Lumpur 57000, Selangor, Malaysia; 4 Department of Chemistry, Central University of Karnataka, Gulbarga 585367, Karnataka, India; 5 Department of Pharmacology, Faculty of Medicine, Bioscience & Nursing, MAHSA University, Jln SP 2, Bandar Saujana Putra, Jenjarom 42610, Selangor, Malaysia; 6 Division of Cellular Medicine, School of Medicine, Jacqui Wood Cancer Centre, University of Dundee, Dundee DD1 4HN, UK; 7 Departments of Medicine and Pharmacology and Molecular Sciences, School of Medicine, Johns Hopkins University, Baltimore, MD 21205, USA; 8 Principal Research Scientist—In Vitro/In Vivo DMPK, Charles River Laboratories Edinburgh Ltd., Tranent EH33 2NE, UK

**Keywords:** 6-shogaol, *Zingiber officinale*, thiophene derivatives, NRF2, anti-inflammatory activity, microsomal stability, molecular docking

## Abstract

6-shogaol is a natural and the most potent bioactive vanilloid in dried *Zingiber officinale* rhizomes. Many scientific studies have reported the diverse biological activities of 6-shogaol. However, the major drawback of 6-shogaol is its instability at room temperature. We synthesised new shogaol thiophene compounds (STCs) by replacing the pentyl group in the sidechain with thiophene derivatives. The STCs were tested for their nuclear factor erythroid 2-related factor 2 (NRF2) activation ability in murine hepatoma cells (Hepa1c1c-7) by determining their NAD(P)H quinone oxidoreductase 1 (NQO1) inducing ability and expression of NRF2-associated antioxidant genes. The anti-inflammatory activity of STCs was determined in *Escherichia coli* lipopolysaccharide (LPS*_Ec_*)-stimulated NR2-proficient and -silenced mouse microglial cells (BV-2) by measuring the inflammatory markers, cytokines, and mediators. The modes of action (interacting with the Kelch domain of KEAP1, covalent bonding with cysteines of KEAP1, and inhibition of GSK-3β enzyme activity) of NRF2 activation by STCs were determined using commercially available kits. The in vitro metabolic stability of the STCs in liver microsomes (humans, rats, and mice) was also investigated. The molecular docking and molecular dynamics studies were conducted to identify the binding poses, stability, and molecular interactions of the STCs in the binding pockets of Kelch and BTB domains of KEAP1 and GSK-3β enzyme. The new STCs were synthesised in good yields of > 85%, with a purity of about 95%, using a novel synthesis method by employing a reusable proline–proline dipeptide catalyst. The STCs are more potent than 6-shogaol in activating NRF2 and reducing inflammation. The nature of substituents on thiophene has a profound influence on the bioactivity of the STCs. Phenylthiophene STC (STC5) is the most potent, while thiophenes containing electron-withdrawing groups showed weaker bioactivity. The bioactivity of 6-shogaol is in the micromolar range, whereas STC5 showed bioactivity in the sub micromolar range. The STCs showed anti-inflammatory effects via NRF2-dependent and NRF2-independent mechanisms. The STCs improved NRF2 activity through multiple (KEAP1-independent and -dependent) mechanisms. The STCs showed decreased reactivity with thiols than 6-shogaol and thus may possess fewer side-effects than 6-shogaol. The STCs were more metabolically stable than 6-shogaol.

## 1. Introduction

**Figure 1:**
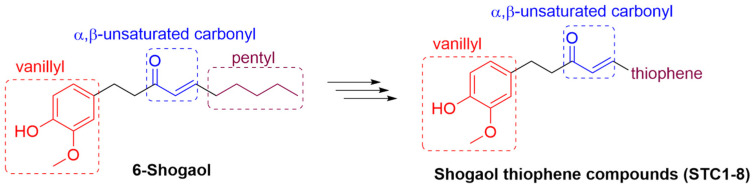
Chemical structure of 6-shogaol and design of shogaol-thiophene compounds (STCs).

6-shogaol (1-(4-hydroxy-methoxyphenyl)-4-decen-one, Figure 1) is a principal vanilloid in dried rhizomes of *Zingiber officinale* (commonly known as ginger). Our group has reported that 6-shogaol is the most potent antioxidant, anti-inflammatory, and anticancer agent among natural vanilloids [1,2,3]. Recent review articles have highlighted the diverse biological activities of 6-shogaol, including metabolic, inflammatory, and neurodegenerative diseases [4,5,6]. Many recent scientific studies have shown that 6-shogaol exhibits potent anti-inflammatory and antioxidant activities in various in vitro and in vivo experimental models that contribute to neuroprotective activity [7], antiviral (SARS-CoV2) activity [8], anticancer activity [9], inhibition of chemotherapy-induced nausea and vomiting [10], treatment of osteoarthritis [11], treatment of rheumatoid arthritis [12], and reducing obesity [13]. 6-shogaol’s biological activities are mediated through various mechanisms, including nuclear factor erythroid 2-related factor 2 (NRF2) activation [14,15]. NRF2 is a master regulator of more than 250 genes that confer antioxidant, anti-inflammatory, cytoprotective, and detoxification effects. The half-life of NRF2 under normal physiological conditions is 20–30 min. NRF2 undergoes proteasomal degradation via binding with Kelch-like ECH-associated protein 1 (KEAP1). There are three functional domains in KEAP1: (1) bric-a-brac (BTB) homodimerisation domain, (2) intervening region (IVR), and (3) C-terminal Kelch domain with a double glycine repeat (DGR). There are two binding units in NRF2, (1) the DLG motif and (2) the ETGE motif, which bind with the Kelch domains of the KEAP1 dimer. Our team and others have extensively reviewed the potential therapeutic benefits of NRF2 activation [16,17,18,19,20,21]. Recent reviews have highlighted the recent updates on NRF2 activators (phytochemicals, semisynthetic, and synthetic), and many researchers across the globe are still looking for novel scaffolds for NRF2 activation [22,23,24,25,26,27,28,29,30]. NRF2 activators are broadly categorised into electrophilic and non-electrophilic activators. The electrophilic NRF2 activators react with thiols (-SH) of KEAP1 cysteine residues. The non-electrophilic activators disrupt the interactions between NRF2 and KEAP1 at the DLG or ETGE motif. The electrophilic NRF2 activators produce “off-target” effects because they can bind to the cysteine-containing proteins. The majority of the phytochemicals, including 6-shogaol, are electrophilic activators. Many reports have claimed that sulforaphane is the most potent NRF2 activator, but it is electrophilic. It is worth noting that one study has reported that 6-shogaol is more potent than sulforaphane in NRF2 activation [31] and obtained a US patent (US10363230B2) on 6-shogaol derivatives as NRF2 activators.

6-shogaol’s chemical structure can be categorised as consisting of three moieties: (1) vanillyl (4-hydroxy-3-methoxybenzyl), (2) α,β-unsaturated carbonyl, and (3) pentyl. The vanillyl and α,β-unsaturated carbonyl moieties are important pharmacophoric features to retain their biological activity [31,32,33]. The α,β-unsaturated carbonyl groups react with KEAP1 cysteines, and the reactivity depends on the steric and electronic parameters of the substituents [34]. There are conflicting reports on the influence of alkyl chain length in gingerols and shogaols on their biological activities [35]. As the alkyl chain length increases, the number of rotatable bonds and conformers increases, thus influencing the compounds’ potency and bioactivity [36]. Organosulfur compounds (e.g., sulforaphane) are known to produce potent NRF2 activity [37,38,39]. We hypothesise that replacing the pentyl sidechain in 6-shogaol with thiophene analogues would improve the NRF2 activity and reduce the reactivity of α,β-unsaturated carbonyl with KEAP1 cysteines. In this study, we synthesised eight novel shogaol thiophene compounds (STCs) (the design of STCs is shown in Figure 1) via a novel synthesis method (using a reusable catalyst). Their bioactivity was investigated using well-established in vitro test models: (1) NRF2 activity in mouse hepatoma cells (Hepa1c1c-7), (2) anti-inflammatory activity in mouse microglial cells (BV-2), (3) “Prochaska” microtiter plate bioassay for inducers of NAD(P)H quinone oxidoreductase 1 (NQO1), (4) qPCR studies for gene expression, and (5) commercially available kits to measure the thiol content, NRF2-KEAP1 inhibition, cytokines expression, and glycogen synthase kinase-3β (GSK-3β). Liver hepatoma cells (Hepa-1c1c7) were selected to study the NRF2 activity because these cells were reported to mimic animal tissues when responding to various chemoprotective agents [40]. Furthermore, Hepa-1c1c7 cells exhibit the characteristics of normal tissues, responsive to stringent environmental nutrition and hormonal factors, mutants defective in the Ah receptor, and highly inducible AHH and Cytochrome P450 [40]. Microglial cells (BV-2) play a crucial role in neurodegenerative disorders and have been used as an experimental model to screen the compounds for neuroprotective activity [41]. Scientific literature reported that lipopolysaccharide (LPS) could induce microglia activation and subsequently initiate the expression of inflammatory mediators such as TNF-α, IL-1β, IL-6, and PGE2 [7,42]. Therefore, BV-2 cells were selected to study the anti-inflammatory effect of STCs. The shogaol thiophene compounds (STCs) showed improved NRF2 and anti-inflammatory effects with reduced reactivity to cysteines via multiple molecular pathways. The interactions between the STCs and the active sites of molecular targets were predicted using molecular docking studies (Schrödinger Drug Discovery Suite 2022-1).

## 2. Materials and Methods

### 2.1. Synthesis of Shogaol Thiophene Compounds (STCs)

**Figure 2:**
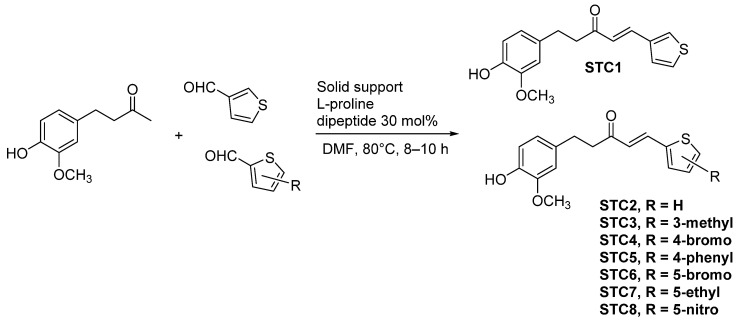
Synthetic scheme for the preparation of shogaol thiophene compounds.

A mixture of solid support proline–proline dipeptide (30 mol%), Vanillylacetone (1 mmol; Sigma Aldrich, St. Louis, MI, USA), and thiophene 3-carbaldehyde (1.2 mmol; Sigma Aldrich, USA) in anhydrous DMF (5 cm^3^; Sigma Aldrich, USA) under inert conditions (nitrogen gas) was stirred at 80 °C for 8 h. The reaction progress was monitored using thin-layer chromatography (TLC, Silica gel 60 F_254_, Merck, St. Louis, MI, USA). After the reaction, the mixture was diluted with cold water and filtered to separate the catalyst. The reaction mixture was then extracted with diethyl ether and removed under reduced pressure. The residue was separated using column chromatography (Silica gel 100–200 mesh, Merck, Rahway, NJ, USA) with a hexane–ethyl acetate mixture to afford pure compounds. The filtered catalyst was reused after drying. All the solvents and reagents were of analytical grade and obtained from Merck. The STCs were characterised using ^1^H, ^13^C nuclear magnetic resonance (NMR; JOEL 400 MHz) and CHN analysis (Thermo Scientific, Waltham, MA, USA). The synthetic scheme is shown in Figure 2. 

### 2.2. Preparation of Test Solutions for Shogaol Thiophene Compounds (STCs)

Accurately weighed amounts of STCs were dissolved in molecular biology grade dimethylsulfoxide (DMSO, Sigma Aldrich, USA) and diluted with phosphate-buffered saline (PBS, pH 7.4; Sigma Aldrich, USA) to prepare the desired concentrations of test solutions. In all cell-based experiments, the effective concentration of DMSO is not more than 0.1%.

### 2.3. Cell Culture

Murine hepatoma cells (Hepa-1c1c7) and murine microglial (BV-2) were used in this experiment. Hepa-1c1c7 was purchased from American Type Culture Collection (ATCC, Manassas, VA, USA), and the cells were cultured and maintained in Alpha minimum essential medium without nucleosides (α-MEM; Gibco, Grand Island, NY, USA) supplemented with 10% foetal bovine serum (FBS; Tico Europe, Amstelveen, Netherlands) and 1% penicillin–streptomycin (100 U/mL penicillin and 100 µg/mL streptomycin). 

BV-2 cells were purchased from Elabscience (Houston, TX, USA) and cultured and maintained in Minimum Essential Medium (MEM; Gibco, Grand Island, NY, USA) supplemented with 10% foetal bovine serum (FBS; Tico Europe, Netherlands) and 1% penicillin–streptomycin (100 U/mL penicillin and 100 µg/mL streptomycin). The cells were maintained in a 37 °C and 5% CO_2_ incubator. Cells from 10 to 20 passages were used for subsequent studies.

### 2.4. Cytotoxicity of Shogaol Thiophene Compounds (STCs)

The safe dose of the test compounds on murine hepatoma (Hepa1c1c-7) and mouse microglial cells (BV-2) was assessed by determining their cytotoxicity using Vybrant^®^ MTT Cell Proliferation Assay Kit (Thermo Fisher Scientific, USA) following the manufacturer’s protocol. In the case of BV-2 cells, the cytotoxicity of the test compounds was assessed together with *E. coli* lipopolysaccharide (LPS*_Ec_*), 200 ng/mL. The cell density was 5 × 10^5^ cells/well (in a 96-well plate), and they were preincubated for 24 h. The test compounds (10, 5, 2.5, 1.25, and 0.625 μM) were then added to each well (in triplicate) and incubated for another 24 h. The solution’s absorbance was measured using Spectramax (Molecular Devices, San Jose, California, USA) M3 microplate reader. The cell viability was calculated using the following formula, and the results are presented as mean ± SD.

$$Percent\;cell\;viability\;\left(\%\right)=\left(\frac{A_{treated\;cells}-A_{blank}{}_{\;}}{A_{untreated\;cells}-A_{blank}}\right)\times 100\%$$

### 2.5. “Prochaska” Microtiter Plate Bioassay

The effect of shogaol thiophene compounds (STCs) on NRF2 activation is determined by measuring the NAD(P)H quinone oxidoreductase 1 (NQO1) inducer activity, as reported in our previous studies [40]. The mouse hepatoma cells (Hepa-1c1c7, 1 × 10^4^ per well) were incubated with STCs (10, 1, 0.1, 0.01, and 0.001 µM) for 48 h. The cells were lysed using digitonin (0.8 mg/mL and 2 mM EDTA; Acros, UK), and the protein concentration was determined using Bradford’s assay kit (Thermo Fisher Scientific, USA). The cell lysate was incubated with a reaction mixture of 0.5 M Tris-Cl buffer (Calbiochem, USA), 7.5 mM FAD (Acros, UK), 150 mM glucose-6-phosphate (Calbiochem, San Diego, CA, USA), 2 U/mL glucose-6-phosphate dehydrogenase (Calbiochem, USA), 50 mM NADP+ (Sigma Aldrich, USA), 25 mM menadione (Sigma Aldrich, USA), and 0.7 mM MTT (Sigma Aldrich, USA) at 37 °C for 5 min. The absorbance was recorded at 610 nm using a microplate reader (Spectramax M3, Molecular Devices, San Jose, CA, USA), and the values were normalised to the total protein content.

### 2.6. Effect of Shogaol Thiophene Compounds on NRF2-Mediated Antioxidant Genes Expression

Hepa1c1c-7 cells were treated with STCs (1 μM) for 48 h, and the total RNA was isolated using QIAzol^®^lysis reagent (Qiagen, Germany). Next, cDNA was synthesised using the ReverTra Ace^®^ qPCR RT Master Mix Kit (Toyobo Research Reagents, Osaka, Japan). Quantitative PCR was performed using THUNDERBIRD^TM^ Next SYBR^®^ qPCR Mix (Toyobo Research Reagents, Osaka, Japan) in CFX96 Touch Real-Time PCR Detection System (Bio-Rad Laboratories, San Jose, CA, USA). The genes tested and the respective primers (purchased from Integrated DNA Technologies, Coralville, IA, USA) used in the study are shown in Table 1. β-actin was used as a reference gene for the normalisation of target gene expression. The 2^−ΔΔCt^ method was used to measure the relative expression of target genes [43].

### 2.7. Effect of Shogaol Thiophene Compounds (STCs) on Inflammation and Thiol Content in BV-2 Cells

The anti-inflammatory activity of the STCs was determined in *E. coli* lipopolysaccharide (LPS*_Ec_;* Sigma Aldrich, USA)-induced inflammation in NRF2-proficient and NRF2 siRNA-transfected mouse microglial (BV-2) cells. The NRF2 was silenced in BV-2 cells using NRF2 siRNA (Santa Cruz Biotechnology, Dallas, TX, USA) in Opti-MEM transfection medium (Thermo Fisher Scientific, USA) using Lipofectamine^TM^ LTX reagent with PLUS^TM^ reagent (Thermo Fisher Scientific, USA) following the manufacturer’s protocol and reported method [44]. The cells (5 × 10^5^ cells/well) were allowed to form a monolayer by incubating them for 24 h and then adding LPS*_Ec_* (200 ng/mL) for 4 h. The STCs (1 µM) were then added and incubated for another 20 h. The nitric oxide (NO) level in cell supernatant was determined using the Griess reagent kit (Thermo Fisher Scientific, USA). The levels of proinflammatory cytokines (IL-1β, IL-6, IFN-γ, and TNF-α) in cell supernatant solutions were determined using commercially available ELISA kits (Abcam, Cambridge, UK). The cell pellets were lysed using Cell Lysis buffer (Thermo Fisher Scientific, Waltham, MA, USA) following the manufacturer’s instructions. The supernatant solution in the wells was replaced with PBS, and cells were detached by scraping, washed with PBS, and centrifuged to collect the cell pellet. The cell pellet was lysed on ice in a cell lysis buffer for half an hour, vortexing at regular intervals. The cell lysate was centrifuged at 15,000 rpm for 15 min at 4 °C. The clear lysate was stored at −80 °C until further use. The protein concentration was determined using Bradford’s assay kit (Thermo Fisher Scientific, USA) following the manufacturer’s instructions. The expression of COX-2, NF-κB p65, NRF2, and HO-1 in cell lysates was determined using commercially available ELISA kits (Abcam, St, Louis, Missouri, USA) following the manufacturer’s protocol. In addition, the thiol content was determined using a thiol quantification assay kit (Abcam, USA).

### 2.8. Effect of Shogaol Thiophene Compounds (STCs) on Glycogen Synthase Kinase 3β (GSK-3β) Enzyme Activity

The effect of STCs on GSK-3β activity was determined using GSK-3β kinase enzyme (Promega, WI, USA) and ADP-Glo^TM^ kinase assay (Promega, USA) following the manufacturer’s protocol and reported method [45]. The STCs were dissolved in the kinase buffer to prepare different concentrations (10, 1, 0.1, 0.01, and 0.001 μM). One microliter of the test compounds was added to each well (384 well plate), followed by the addition of 2 μL of GSK-3β kinase (1 ng), 2 μL of the GSK-3β substrate (0.4 μg), and 2 μL of the ATP (25 μM), and incubated for 60 min. Then, 5 μL of ADP-Glo^TM^ reagent was added and incubated further for 40 min. Then 10 μL of kinase detection reagent was added and incubated for 30 min. The luminescence was recorded using a SpectraMax^®^L microplate reader (Molecular Devices, San Jose, CA, USA). The IC_50_ (concentration of the test compound required to produce half-maximal kinase activity) from dose-response curves using non-linear regression analysis using GraphPad Prism (9.5.0; San Diego, CA, USA) software.

### 2.9. Liver Microsomal Stability Studies

The liver microsomes of humans, rats, and mice were purchased from Merck (human, #M0567; rat, #M9066; mouse, #M9441). The metabolic stability of 6-shogaol and STCs (1 μM effective concentration; 5 μL) was mixed with liver microsomes (0.5 mg mL^−1^; 445 μL) in a microcentrifuge tube. Verapamil (0.5 μM) was used as the positive control. The microsome and compound mixture were shaken on a plate shaker for 5 min to ensure homogeneity. NADPH (1 mM; 50 μL) was added to the mixture to start the metabolic reaction. The reaction mixture was incubated at 37 °C for 0, 3, 6, 15, 30, 45, and 60 min. The mixture (20 μL) was sampled and quenched with 180 μL of acetonitrile with internal standard (Donepezil 50 ng/mL) to terminate the metabolic reaction at each time point. Ultrapure water (80 μL) was added, and contents in the microcentrifuge tubes were centrifuged at 3800 rpm at 4 °C for 10 min. Next, the concentration of 6-shogaol and STCs found in the supernatant layer was quantified using Agilent 1290 coupled with Q-TOF. Each set of experiments was repeated three times in human, rat, and mouse liver microsomes. The concentration of the compounds versus time was plotted to determine the elimination rate constant (K_el_). The intrinsic clearance (Cl_int_) and half-life (t_1/2_) were calculated using the following equations. The intrinsic clearance was calculated with scaling factors of microsomal protein (52.5 mg microsomal protein/g liver).

$${\mathrm{Cl}}_{\mathrm{int}}=\left(\frac{K_{\mathrm{el}}}{0.5}\right)\times 52.5\;\;t_{1/2}=\frac{0.693}{K_{\mathrm{el}}}$$

### 2.10. Fluorescence Polarisation Assay

The KEAP1-Nrf2 inhibitor screening assay kit (BPS Bioscience, San Diego, California, USA) was used to determine the effect of STCs in inhibiting KEAP1-NRF2 interaction following the manufacturer’s protocol and our reported method [46]. The STC solutions were diluted with the assay buffer, and 5 μL of the STC solutions were incubated at room temperature for 30 min in the peptide mixture containing 0.5 μL NRF2 peptide (1 μM) and 20 μL KEAP1 (15 ng/μL). The fluorescence polarisation (FP) was measured using a microplate reader at λ_ex_ 485 nm and λ_em_ 530 nm. The following equation was used to determine the per cent KEAP1-NRF2 inhibitory effect of the STCs.

$$\mathrm{Keap}1-\mathrm{Nrf}2\;\mathrm{inhbitory}\;\mathrm{activity}\;\left(\%\right)=\left(1-\frac{\mathrm{Relative}\;\mathrm{FP}\;\mathrm{of}\;\mathrm{STC}}{\mathrm{Relative}\;\mathrm{FP}\;\mathrm{of}\;\mathrm{postive}\;\mathrm{control}}\right)\times 100$$

### 2.11. In Silico Studies

The binding poses of STCs and their molecular interactions with amino acid residues in the binding sites of KEAP1 Kelch domain and GSK-3β were determined using Fast Rigid Exhaustive Docking (FRED) 4.2.0.1 (OpenEye Scientific Software, Santa Fe, NM, USA) [47,48]. The crystal structures of the (1) KEAP1 Kelch domain complexed with *N*,*N*′-naphthalene-1,4-diylbis(4-methoxybenzenesulfonamide) (PDB ID: 4IQK) with a resolution of 1.97 A° [49] and (2) GSK-3β complexed with (*Z*)-1H,1′H-[2,3′]bi-indolylidene-3,2′-dione-3-oxime (PDB ID: 1Q41) with a resolution of 2.10 A° [50] were used for molecular docking studies. The “Make Receptor application 4.2.0.1” (OpenEye Scientific Software, USA) [51] with default setting was used to generate the grid with the default setting around the inbound ligand. The OMEGA 4.2.1.1 (OpenEye Scientific Software, Santa Fe, NM, USA) [52] application was used to generate all the possible conformers of the compounds. The ability of test compounds to form a covalent binding with cysteine thiol was predicted using the “Covalent docking” module in Schrödinger Drug Discovery Suite (2022-2, Schrödinger, Inc., New York, NY, USA) with default settings. The crystal structure of KEAP1’s BTB (Broad-Complex, Tramtrack and Bric a Brac) complexed with TX64063 (PDB ID: 5DAF) with a resolution of 2.37 A° [53] was used for covalent docking studies [54]. In addition, docking scores and binding energies of the compounds in the binding pockets of KEAP1’s Kelch domain (PDB ID: 4IQK) and GSK-3β (PDB ID: 1Q41) were predicted using GLIDE [55] and prime MM-GBSA applications in Schrödinger Drug Discovery Suite (2022-2) with default settings. Molecular dynamics (MD) simulations (with default settings) were performed with the Desmond package in Schrödinger Drug Discovery Suite to determine the stability of the most potent compound (STC5) and 6-shogaol in the binding pockets of 4IQI and 1Q41. The protein–ligand complexes were immersed in a TIP3P (transferable intermolecular potential with 3 points) water box (10 Å). Counter ions (Na^+^ and Cl^−^ ions) were added to neutralise the charges. The MD was performed in the NPT ensemble (at 300 K and 1.63 bar) for 100 ns. The OPLS-3e force field was used. The root means square deviation (RMSD) of ligand and protein over the 100 ns MD trajectory were recorded.

### 2.12. Statistical Analysis

The statistical analysis of the results was carried out using GraphPad Prism 9.5.0, and results were presented as mean ± SD. The statistical significance (*, *p* < 0.05 (*), *p* < 0.01 (**), *p* < 0.001 (***), and *p* < 0.0001 (****)) was calculated using Brown–Forsythe and Welch ANOVA tests, followed by the DunnetteT3 test. 

## 3. Results

### 3.1. Characterisation of STCs

#### 3.1.1. STC1: (E)-5-(4-Hydroxy-3-methoxyphenyl)-1(thiophen-3-yl)pent-1-en-3-one (Scheme 1)

^1^H NMR (400 MHz, CDCl_3_): δ 2.81(s, 3H, 8″-OCH_3_), 3.71(dd, 4H, 4,5-CH_2_), 5.14(brs, 1H, 7″-OH), 6.44(d, *J* = 16.04 Hz, 1H, 2″-CH), 6.59(t, *J* = 15.84 Hz, 2H, 5″,6″-CH), 6.73 (d, *J* = 7.96 Hz, 1H, 1′-CH), 7.18 (d, *J* = 8.12 Hz, 2H, 3′,4′-CH), 7.37(d, *J* = 4.06 Hz, 1H, 1-CH), 7.43 (d, *J* = 16.2 Hz, 1H, 2-CH)

^13^CNMR (100 MHz, CDCl_3_): δ 29.96(5-CH_2_), 42.66(4-CH_2_), 55.90(8″-OCH_3_), 111.34(2″-CH), 114.54(5″-CH), 120.84(6″-CH), 125.29(4′-HC), 126.10(3′-CH), 127.80(1′-CH), 128.86(2′-C), 133.14(2-CH), 136.29(1″-C), 138.29(C-1), 144.60(4″-C), 146.89(3″-C), 199.98(3-CO).

Molecular Formula: C_16_HO_3_S

Elemental Analysis shown: C, 66.64; H, 5.59; O, 16.65; S, 11.12

Elemental Analysis found: C, 66.69; H, 5.55; O, 16.61; S, 11.13

Percentage yield: 87%

#### 3.1.2. STC2: (E)-5-(4-Hydroxy-3-methoxyphenyl)-1-(thiophen-2-yl)pent-1-en-3-one (Scheme 2)

^1^H NMR (400 MHz, CDCl_3_): δ 2.75 (s, 3H, 8″-CH_3_), 3.63 (d, 4H, 4,5-CH_2_), 5.18(brs, 1H, 7″-OH), 6.38(d, *J* = 15.76 Hz, H-2″-CH), 6.53(d, *J* = 12.8 Hz, 2H, 5″,6″-CH), 6.71(d, *J* = 10.6 Hz, 2H, 2′,4′-CH), 6.85(dd, 1H, 3′-CH), 7.23(d, *J* = 8.14 Hz, 1H, 1-CH), 7.51(d, *J* = 15.72 Hz, 1H, 2-CH)

^13^CNMR (100 MHz, CDCl_3_): δ 29.42(5-CH_2_), 42.89(4-CH_2_), 55.90(8″-OCH_3_), 111.42(2″-CH), 114.58(5″-CH), 120.77(6″-CH), 124.68(3′-CH), 128.61(2′-CH), 129.04(4′-CH), 131.81(1-CH), 132.99(2-CH), 135.28(1″-C), 139.81(1-C’), 144.25(4″-C), 146.87(3″-C), 199.27(3-CO)

Chemical formula: C_16_H_16_O_3_S

Elemental analysis shown: C, 66.64; H, 5.59; O, 16.65; S, 11.12

Elemental analysis found: C, 66.69; H, 5.55; O, 16.61; S, 11.13

Percentage yield: 89%

#### 3.1.3. STC3: (E)-5-(4-Hydroxy-3-methoxyphenyl)-1-(3-methylthiophen-2-yl)pent-1-en-3-one (Scheme 3)

^1^H NMR (400 MHz, CDCl_3_): δ 2.27(s, 3H, 8″-OCH_3_), 2.88(dd, 4H, 4,5-CH_2_), 3.84(s, 3H, 5′-CH_3_), 5.93(brs, 1H, 7″-OH), 6.41(d, *J* = 16.04 Hz, 1H, 2″-CH), 6.71(d, *J* = 8.12 Hz, 1H, 4′-CH), 6.68(d, *J* = 10.04 Hz, 1H, 3′-CH), 6.82(t, *J* = 3.2 Hz, 2H, 5″,6″-CH), 7.22(d, *J* = 5.04 Hz, 1H, 1-CH), 7.72(d, *J* = 16.04 Hz,1H, 2-CH)

^13^CNMR (100 MHz, CDCl_3_): δ 15.52(5′-CH), 30.06(5-CH_2_), 43.31(4-CH_2_), 55.90(8″-OCH_3_), 111.33(2″-CH), 114.55(5″-CH), 120.55(6″-CH), 123.77(1′-C), 127.45(1-CH), 131.42(1″-C), 133.11(2-CH), 133.84(4′-CH), 133.90(3′-CH), 142.42(2′-C), 144.05(4″-C), 146.62(3″-C), 199.18(3-CO)

Chemical formula: C_17_H_18_O_3_S

Elemental analysis shown: C, 67.52; H, 6.00; O, 15.87; S, 10.60

Elemental analysis found: C, 67.55; H, 6.04; O, 15.84; S, 10.58

Percentage yield: 86%

#### 3.1.4. STC4: (E)-5-(4-Hydroxy-3-methoxyphenyl)-1-(4-bromothiophen-2-yl)pent-1-en-3-one (Scheme 4)

^1^H NMR (400 MHz, CDCl_3_): δ 2.88(s, 3H, 8″-OCH_3_), 3.75(s, 4H, 4,5-CH_2_), 5.62(brs, 1H, 7″-OH), 6.42(d, *J* = 10.4 Hz, 1H, 5″-CH), 6.61(m, 1H, 2″-CH), 6.63(d, *J* = 10.4 Hz, 1H, 6″-CH), 6.73(d, *J* = 7.96 Hz, 1H, 2′-CH), 7.04(s, 1H, 4′-CH), 7.16(s, 1H, 1-CH), 7.42(d, *J* = 15.88 Hz, 1H, 2-CH)

^13^CNMR (100 MHz, CDCl_3_): δ 29.49 (6″-CH), 43.18(5″-CH), 55.90(8″-OCH_3_), 109.10(4-CH_2_), 111.16(2″-CH), 114.48(5″-CH), 120.51(6″-CH), 125.70(2′-CH), 132.91(5-CH_2_), 133.00(1″-C), 133.58(2″-CH), 140.59(2-CH), 143.96(4″-C), 144.01(3″-C), 146.52(1″-CH), 198.74 (3-CO)

Chemical formula: C_16_H_15_BrO_3_S

Elemental analysis shown: C, 52.33; H, 4.12; Br, 21.76; O, 13.07; S, 8.73 

Elemental analysis found: C, 52.36; H, 4.15; Br, 21.72; O, 13.07; S, 8.76

Percentage yield: 86%

#### 3.1.5. STC5: (E)-5-(4-Hydroxy-3-methoxyphenyl)-1-(4-phenylthiophen-2-yl)pent-1-en-3-one (Scheme 5)

^1^H NMR (400 MHz, CDCl_3_): δ 2.85(s, 3H, 8″-OCH_3_), 3.78(dd, 4H, 4,5-CH_2_), 5.49(brs, 1H, 7″-OH), 6.47(d, *J* = 15.76 Hz, 1H, 2″-CH), 6.65(dd*, J_1_ =* 1.8 Hz*, J_2_ =* 2.76 Hz*,* 2H, 5″,6″-CH), 6.76(d, *J* = 7.92 Hz, 1H, 1-CH), 7.24(t, *J* = 7.4 Hz, 1H, 8′-CH), 7.33(t, *J* = 7.28Hz, 2H, 7′,9′-CH), 7.40(s, 1H, 2-CH), 7.46(d, *J* = 4.08 Hz, 2H, 6′,10′-CH), 7.48(d, *J* = 1.36 Hz, 1H, 2′-CH), 7.61(d, *J* = 15.8Hz, 1H, 4′-CH).

^13^CNMR (100 MHz, CDCl_3_): δ 29.94 (5-CH_2_), 43.14(4-CH_2_), 55.91(8″-OCH_3_), 111.14(2″-CH), 114.38(5″-CH), 120.85(4′-CH), 123.49(6″-CH), 125.05(6′,10′-CH), 126.27(8′-CH), 127.72(7′,9′-CH), 128.97(2′-CH), 130.41(1-CH), 133.09(2-CJ), 134.88(1″-C), 135.04(5′-C), 140.40(1′-C), 143.46(3′-C), 143.96(4″-C), 146.45(3″-C), 198.90(3-CO)

Chemical formula: C_22_H_20_O_3_S

Elemental analysis shown: C, 72.50; H, 5.53; O, 13.17; S, 8.80 

Elemental analysis found: C, 72.47; H, 5.55; O, 13.14; S, 8.83

Percentage yield: 87%

#### 3.1.6. STC6: (E)-5-(4-Hydroxy-3-methoxyphenyl)-1-(5-bromothiophen-2-yl)pent-1-en-3-one (Scheme 6)

^1^H NMR (400 MHz, CDCl_3_): δ 2.75(s, 3H, 8″-OCH_3_), 3.76(s, 4H, 4,5-CH_2_), 6.32(d, *J* = 3.68 Hz, 1H, 2″-CH), 6.49(d, *J* = 3.44 Hz, 1H, 5″-CH), 6.61(m, 1H, 6″-CH), 6.65(d, *J* = 1.84 Hz, 1H, 1-CH), 6.74(dd, *J_1_* = 2.92 Hz *J_2_* = 4.32 Hz, 2H, 2′,3′-CH), 7.12(d, *J* = 15.72 Hz, 1H, 2-CH).

^13^CNMR (100 MHz, CDCl_3_): δ 29.88 (5-CH_2_), 43.59(4-CH_2_), 55.90(8″-OCH_3_), 109.10(2″-CH), 111.77(5″-CH), 114.44(4′-C), 117.87(6″-CH), 120.74(3′-CH), 120.81(2′-CH), 123.37(1-CH), 127.51(2-CH), 132.98(1″-C), 143.94(1′-C), 146.49(4″-C), 152.92(3″-C), 198.93(3-CO)

Chemical formula: C_16_H_15_BrO_3_S

Elemental analysis shown: C, 52.33; H, 4.12; Br, 21.76; O, 13.07; S, 8.73

Elemental analysis found: C, 52.36; H, 4.15; Br, 21.72; O, 13.07; S, 8.76

Percentage yield: 88%

#### 3.1.7. STC7: (E)-5-(4-Hydroxy-3-methoxyphenyl)-1-(5-ethylthiophen-2-yl)pent-1-en-3-one (Scheme 7)

^1^H NMR (400 MHz, CDCl_3_): δ 1.31(t, *J* = 6.84 Hz, 3H, 8″-OCH_3_), 2.84(q, *J_1_* = 7.32 Hz, *J_2_* = 15.12 Hz, 2H, 5′-CH_2_), 2.89(dd, 4H, 4,5-CH_2_), 3.85(s, 3H, 6′-CH_3_), 5.57(brs, 1H, 7″-OH), 6.41(d, *J* = 16.04 Hz, 1H, 2″-CH), 6.71(m, 3H, 5″,6″,3′-CH), 6.82(d, *J* = 7.76 Hz, 1H, 2′-CH), 7.07(d, *J* = 3.64 Hz, 1H, 1-CH), 7.58(d, *J* = 16.04 Hz, 1H, 2-CH)

^13^CNMR (100 MHz, CDCl_3_): δ 15.69 (5-CH_2_), 24.01(4-CH_2_), 30.24(8″-OCH_3_), 43.01(5′-CH), 55.96(6′-CH), 111.20(2″-CH), 114.42(5″-CH), 120.89(4′-C), 123.65(6″-CH), 125.02(3′-CH), 132.50(2′-CH), 133.28(1-CH), 135.82(2-CH), 137.46(1″-C), 143.97(1′-C), 146.51(4″-C), 152.34(3″-C), 199.17(3-CO)

Chemical formula: C_18_H_20_O_3_S

Elemental analysis shown: C, 68.33; H, 6.37; O, 15.17; S, 10.13 

Elemental analysis found: C, 68.36; H, 6.34; O, 15.15; S, 10.17

Percentage yield: 88%

#### 3.1.8. STC8:(E)-5-(4-Hydroxy-3-methoxyphenyl)-1-(5-nitrothiophen-2-yl)pent-1-en-3-one (Scheme 8)

^1^H NMR (400 MHz, CDCl_3_): δ 2.78(m, 4H, 4,5-CH_2_), 3.81(s, 3H, 8″-OCH_3_), 5.77(brs, 1H, 7″-OH), 6.61(t, *J* = 13.32 Hz, 2H, 5″,6″-CH), 6.65(s, 1H, 2″-CH), 6.72(dd, *J_1_*= 2.96Hz, *J_2_* = 8.4 Hz, 1H, 3′-CH), 7.11(d, *J* = 4.2 Hz, 1H, 2′-CH), 7.42(d, *J* = 15.92 Hz, 1H, 1-CH), 7.75(d, *J* = 1.76 Hz, 1H, 2-CH)

^13^CNMR (100MHz, CDCl_3_): δ 29.66(5-CH_2_), 47.44(4-CH_2_), 55.97(8″-OCH_3_), 111.20(2″-CH), 114.50(5″-CH), 120.87(6″-CH), 128.62(3′-CH), 129.23(1-CH), 129.63(2-CH), 132.69(1″-C), 132.96(2′-CH), 144.08(1′-C), 145.99(4″-C), 146.58(3″-C), 152.21(4′-C), 198.29(3-CO)

Chemical formula: C_16_H_15_NO_5_S

Elemental analysis shown: C, 57.65; H, 4.54; N, 4.20; O, 24.00; S, 9.62 

Elemental analysis found: C, 57.59; H, 4.56; N, 4.17; O, 24.05; S, 9.66

Percentage yield: 89%

### 3.2. Cytotoxicity of STCs

The cytotoxic effect of STCs on Hepa-1c1c7 cells is shown in the Supplementary Information (Figure S18A). The 6-shogaol at 5 and 10 μM showed cell viability of less than 90%. The compound STC-8 at 2.5, 5, and 10 μM showed cell viability of less than 90%. All other compounds showed more than 90% cell viability at all concentrations (10, 5, 2.5, 1.25, and 0.625 μM). The results indicate synthesised STCs, except STC-8, are less cytotoxic than 6-shogaol. 

The anti-inflammatory activity of STCs was investigated using LPS*_Ec_* (200 ng/mL)-challenged BV-2 cells; thus, a safe dose of the STCs (in the presence of LPS*_Ec_*) on BV-2 cells was determined via their cytotoxicity on BV-2 cells. The results are shown in the Supplementary Information (Figure S18B). 6-shogaol plus LPS*_Ec_* showed less than 90% cell viability at concentrations ≥ 5 μM. The combination of STC-8 plus LPS*_Ec_* showed less than 90% cell viability at concentrations ≥ 2.5 μM. All other STCs (in the presence of LPS*_Ec_*) showed less than 90% cell viability at 10 μM. 

### 3.3. Effect of STCs on NQO1 Induction and Expression of NRF2-Associated Genes in Hepa-1c1c7 Cells

**Figure 3:**
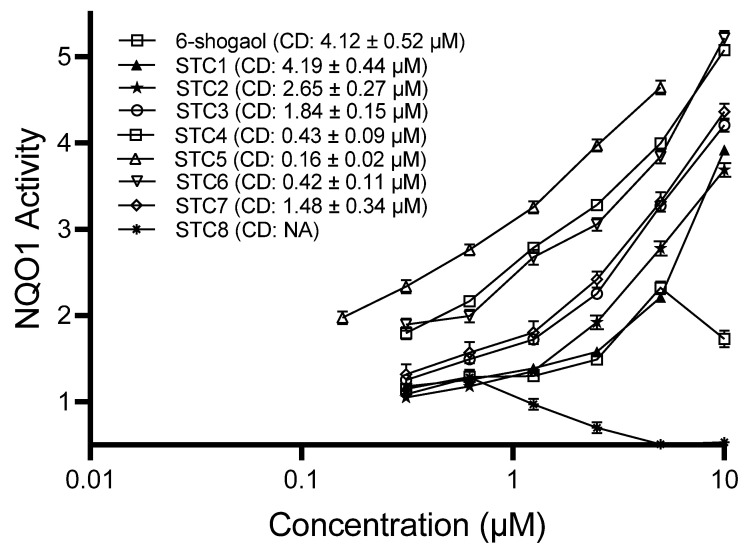
Dose-response curve of 6-shogaol and STCs on NQO1 induction in Hepa-1c1c7 cells.

Figure 3 shows the dose-dependent (10, 5, 2.5, 1.25, and 0.625 μM) NQO1-inducing ability of STCs. The 6-shogaol’s CD (concentration required to double the NQO1 level) is 4.12 ± 0.52 μM, and 6-shogaol were toxic to the cells at 10 μM. All the synthesised STCs (except STC1 and STC8) were more active than 6-shogaol. The STC8 did not show the CD value and was toxic at concentrations greater than 1 μM. The STC1 containing 3-thiophene moiety showed activity similar to 6-shogaol, whose CD value is 4.19 ± 0.44 μM. The compounds containing 2-thiophene analogues (STC2, STC3, STC4, STC5, STC6, and STC7) are more potent than 6-shogaol, with CD values of 2.65 ± 0.27, 1.84 ± 0.15, 0.43 ± 0.09, 0.06 ± 0.02, 0.42 ± 0.11, and 1.48 ± 0.34 μM, respectively. The STC5 (containing 4-phenyl-2-thiophene) is the most potent. The decreasing order of potency (based on CD values, lower CD means more potent) of STCS in inducing NQO1 are STC5 > (STC4~STC6) > STC7 > STC3 > STC 2 > (STC1~6-shogaol). 

**Figure 4:**
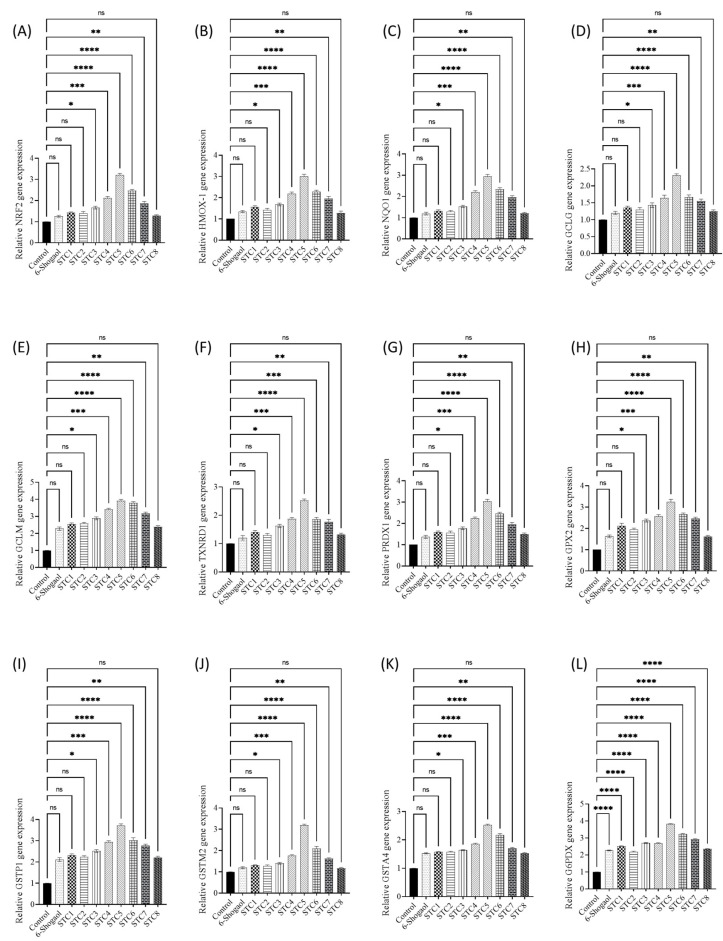
The effect of STCs and 6-shogaol on the expression of representative antioxidant genes (**A**) nuclear factor erythroid 2–related factor 2 (NRF2), (**B**) heme oxygenase-1 (HMOX1), (**C**) human NADPH quinone oxidoreductase (NQO1), (**D**) GCLG, (**E**) glutamate-cysteine ligase modifier subunit (GCLM), (**F**) thioredoxin reductase 1 (TXNRD1), (**G**) Peroxiredoxin-1 (PRDX1), (**H**) glutathione peroxidase 2 (GPX2), (**I**) glutathione S-transferases P1 (GSTP1), (**J**) glutathione S-transferase Mu 2 (GSTM2), (**K**) g lutathione S-transferase alpha 4 (GSTA4), (**L**) glucose-6-phosphate dehydrogenase X-linked (G6PDX) in Hepa-1c1c7 cells. The expressions of these genes were found to be significantly upregulated by the compound STC5. The experiment was performed in triplicate and each experiment was repeated twice. ^ns^, not significant; *p* < 0.05 (*); *p* < 0.01 (**); *p* < 0.001 (***); and *p* < 0.0001 (****)).

The qPCR studies were carried out to determine the effect of 6-Shogoal and STCs (at 1 μM) on the expression of the NRF2 gene and its downstream representative eleven antioxidant genes (HMOX1, NQO1, GCLC, GCLM, TXNRD1, PRDX1, GPX2, GSTP1, GSTM2, GSTA1, and G6PDX). The gene expression results are shown in Figure 4. All the compounds (6-shogaol and STCs) have upregulated the expression of all the genes (NRF2 and its associated antioxidant genes). STC5 at 1 μM showed the maximum activity. STC1’s activity is almost similar to that of 6-shogaol. STC8 showed lower activity than 6-shogaol. The compounds STC4 and STC6 showed similar activity. The foldchange (compared to control) in the upregulation of genes expression by STC5 compared to 6-shogaol (fold change values are shown in parenthesis) are as follows: (1) NRF2, 3.21 ± 0.08 (1.26 ± 0.05); (2) HMOX1, 3.01 ± 0.10 (1.34 ± 0.05); (3) NQO1, 2.94 ± 0.01 (1.20 ± 0.06); (4) GCLC, 2.31 ± 0.05 (1.20 ± 0.05); (5) GCLM, 3.91 ± 0.09 (2.28 ± 0.10); (6) TXNRD1, 2.52 ± 0.06 (1.20 ± 0.08); (7) PRDX1, 3.04 ± 0.09 (1.37 ± 0.07); (8) GPX2 3.25 ± 0.11 (1.63 ± 0.06); (9) GSTP1, 3.71 ± 0.09 (2.12 ± 0.09); (10) GSTM2, 3.19 ± 0.05 (1.19 ± 0.05); (11) GSTA4, 2.52 ± 0.03 (1.53 ± 0.02); and (12) G6PDX, 3.83 ± 0.02 (2.27 ± 0.03).

### 3.4. Effect of STCs on Anti-Inflammatory Markers and Mediators in BV-2 Cells

**Figure 5:**
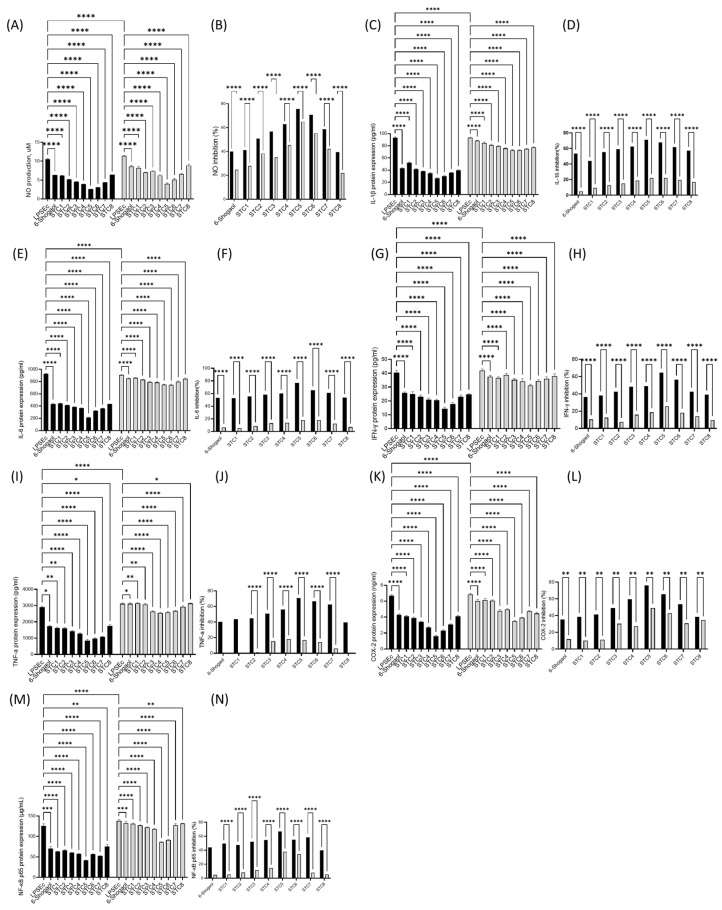
The effect of STCs and 6-shogaol on the production of inflammatory markers namely (**A**) nitric oxide (NO) production and (**B**) percent NO inhibition; cytokines namely (**C**), Interleukin 1-beta (IL-1β) gene expression and (**D**) percent IL-1β inhibition, (**E**) IL-6 gene expression and (**F**) percent IL-6 inhibition, (**G**)interferon-gamma (IFN-γ) and (**H**) percent IFN-γ inhibition, (**I**) tumour necrosis factor-alpha (TNF-α) and (**J**) percent TNF-α inhibition; and mediators namely (**K**) cyclooxygenase-2 (COX-2) and (**L**) percent COX-2 inhibition, (**M**) nuclear factor kappa B (NF-κB) and (**N**) percent NF-κB inhibition in LPS*_Ec_*-challenged in NRF2-proficient and -silenced BV-2 cells. The experiment was performed in triplicate and each experiment was repeated twice. ^ns^, not significant; *p* < 0.05 (*); *p* < 0.01 (**); *p* < 0.001 (***); and *p* < 0.0001 (****)).

The anti-inflammatory activity of STCs (at 1 μM concentration) was assessed by measuring nitric oxide (NO), proinflammatory cytokines (IL-1β, IL-6, IFN-γ, and TNF-α), and inflammatory mediators (COX-2, and NF-κB p65) production in LPS*_Ec_*-stimulated NRF2-Proficient and -silenced BV-2 cells (Figure 5). The ability of NRF2 siRNA to silence NRF2 in BV-2 cells was confirmed by comparing the NRF2 gene levels in control siRNA and NRF2 siRNA transfected BV-2 cells and NRF2-proficient BV-2 cells (the results are shown in the Supplementary Information). 

The effect of 6-shogaol and STCs on NO production is shown in Figure 5A, and the percentage of NO inhibition is shown in Figure 5B. LPS*_Ec_* (200 ng/mL) produced NO in NRF2-proficient BV-2 cells was 10.53 ± 0.22 μM, and in NRF-2 silenced cells, it produced 11.37 ± 0.15 μM NO. All the STCs and 6-shogaol inhibited the NO production. STCs containing 2-thiophene analogues (STC2-7) are more active than 6-shogaol (6.32 ± 0.03 μM in NRF2-proficient BV-2 cells and 8.56 ± 0.36 μM in NRF2-silenced BV-2 cells). STC1 (containing 3-thiophene) and STC8 (containing 5-nitro-2-thiophene) are as active as 6-shogaol. The STC5 is the most active, and NO production was 2.58 ± 0.04 μM in the NRF2-proficient BV-2 cells and 3.0 ± 0.28 μM in NRF2-silenced BV-2 cells. Among the 2-thiophene analogues, STC8 is the least active; NO production was 6.38 ± 0.04 μM in the NRF2-proficient BV-2 cells and 8.86 ± 0.38 μM in NRF2-silenced BV-2 cells. The results (Figure 5B) have shown that the 6-shogaol and STCs have more effect on NO inhibition in NRF2-proficient BV-2 cells than in NRF2-silenced BV-2 cells. 6-shogaol inhibited 40.00% inhibition in NRF2-proficient BV-2 cells and 24.69% in NRF2-silenced BV-2 cells. The most active compound, STC5, inhibited 75.51% NO production in NRF2-proficient BV-2 cells and only 64.81% in NRF2-silenced BV-2 cells. 

The effect of 6-shogaol and STCs on IL-1β production is shown in Figure 5C, and the percentage of IL-1β inhibition is shown in Figure 5D. LPS*_Ec_* (200 ng/mL) produced IL-1β in NRF2-proficient BV-2 cells 93.53 ± 1.10 pg/mL, and in NRF-2 silenced BV-2 cells, it produced 93.23 ± 0.90 pg/mL. All the STCs and 6-shogaol reversed the elevated levels of IL-1β. All the STCs (except STC1 containing 3-thiophene) are more active than 6-shogaol (43.30 ± 0.62 pg/mL in NRF2-proficient BV-2 cells and 88.57 ± 0.55 pg/mL in NRF2-silenced BV-2 cells). The STC5 is the most active, and IL-6 levels were 26.77 ± 0.85 pg/mL in the NRF2-proficient BV-2 cells and 72.50 ± 1.32 pg/mL in NRF2-silenced BV-2 cells. Among the 2-thiophene analogues, STC8 is the least active; IL-1β levels were 39.93 ± 0.74 pg/mL in the NRF2-proficient BV-2 and 77.33 ± 0.93 pg/mL in NRF2-silenced BV-2 cells. The results (Figure 5D) have shown that the 6-shogaol and STCs have more effect on the reversal of elevated IL-6 levels in NRF2-proficient cells than in NRF2-silenced BV-2 cells. 6-shogaol inhibited 53.70%% inhibition in NRF2-proficient BV-2 cells and 5.00% in NRF2-silenced BV-2 cells. The most active compound, STC5, inhibited 71.38% of NRF2-proficient BV-2 cells and only 22.34% in NRF2-silenced BV-2 cells. 

The effect of 6-shogaol and STCs on IL-6 production is shown in Figure 5E, and the percentage of IL-6 inhibition is shown in Figure 5F. LPS*_Ec_* (200 ng/mL) produced IL-6 in NRF2-proficient BV-2 cells, and the amount was 926.33 ± 4.73 pg/mL. In NRF-2 silenced BV-2 cells, it produced 909.67 ± 2.52 pg/mL. All the STCs and 6-shogaol reversed the elevated levels of IL-6. The compounds STC1 and STC8 are equally active as 6-shogaol. All the other STCs (STC 2-7) are more active than 6-shogaol (435.67 ± 7.77 pg/mL in NRF2-proficient BV-2 cells and 852.67 ± 5.69 pg/mL in NRF2-silenced BV-2 cells). The STC5 is the most active, and IL-6 levels were 212.33 ± 6.11 pg/mL in the NRF2-proficient BV-2 cells and 748.67 ± 8.33 pg/mL in NRF2-silenced BV-2 cells. Among the 2-thiophene analogues, STC8 is the least active; IL-6 levels were 431.667 ± 3.06 pg/mL in the NRF2-proficient BV-2 cells and 846.67 ± 11.68 pg/mL in NRF2-silenced BV-2 cells. The results (Figure 5F) have shown that the 6-shogaol and STCs have more effect on the reversal of elevated IL-6 levels in NRF2-proficient BV-2 cells than in NRF2-silenced BV-2 cells. 6-shogaol inhibited 52.97%% inhibition in NRF2-proficient BV-2 cells and 6.27% in NRF2-silenced BV-2 cells. The most active compound, STC5, inhibited 77.08% of NRF2-proficient BV-2 cells and only 17.70% in NRF2-silenced BV-2 cells.

The effect of 6-shogaol and STCs on IFN-γ production is shown in Figure 5G, and the percentage of IFN-γ inhibition is shown in Figure 5H. LPS*_Ec_* (200 ng/mL) produced IFN-γ in NRF2-proficient BV-2 cells, and the amount was 40.44 ± 1.69 pg/mL. In NRF-2 silenced BV-2 cells, it produced 41.91 ±1.10 pg/mL. All the STCs and 6-shogaol reversed the elevated levels of IFN-γ. The compounds STC1 and STC8 are equally active as 6-shogaol. All the other STCs (STC 2-7) are more active than 6-shogaol (25.75 ± 0.72 pg/mL in NRF2-proficient BV-2 cells and 37.52 ± 1.01 pg/mL in NRF2-silenced BV-2 cells). The STC5 is the most active, and IFN-γ levels were 14.34 ± 1.10 pg/mL in the NRF-2 proficient BV-2 cells and 31.19 ± 0.85 pg/mL in NRF2-silenced BV-2 cells. Among the 2-thiophene analogues, STC8 is the least active; IFN-γ levels were 24.63 ± 0.64 pg/mL in the NRF2-proficient BV-2 cells and 37.86 ± 1.69 pg/mL in NRF2-silenced BV-2 cells. The results (Figure 5H) have shown that the 6-shogaol and STCs have more effect on the reversal of elevated IFN-γ levels in NRF2-proficient BV-2 cells than in NRF2-silenced BV-2 cells. 6-shogaol inhibited 36.33% in NRF2-proficient BV-2 cells and 10.48% in NRF2-silenced BV-2 cells. The most active compound, STC5, inhibited 42.73% of NRF2-proficient BV-2 cells and only 25.58% in NRF2-silenced BV-2 cells.

The effect of 6-shogaol and STCs on TNF-α production is shown in Figure 5I, and the percentage of TNF-α inhibition is shown in Figure 5J. LPS*_Ec_* (200 ng/mL) produced TNF-α in NRF2-proficient BV-2 cells was 2907.00 ± 51.00 pg/mL, and in NRF-2 silenced BV-2 cells, it produced 3111.00 ± 51.00 pg/mL. All the STCs and 6-shogaol reversed the elevated levels of TNF-α. The compounds (STC1 and STC8) are equally active as 6-shogaol. All the other STCs (STC 2-7) are more active than 6-shogaol (1734.00 ± 51.00 pg/mL in NRF2-proficient BV-2 cells and 3113.00 ± 55.34 pg/mL in NRF2-silenced BV-2 cells). The STC5 is the most active, and TNF-α levels were 850.00 ± 77.90 pg/mL in the NRF2-proficient BV-2 cells and 2581.00 ± 51.00 pg/mL in NRF2-silenced BV-2 cells. Among the 2-thiophene analogues, STC8 is the least active; TNF-α levels were 1751.00 ± 77.90 pg/mL in the NRF2-proficient BV-2 cells and 3130.33 ± 27.47 pg/mL in NRF2-silenced BV-2 cells. The results (Figure 5J) have shown that the 6-shogaol and STCs have more effect on the reversal of elevated TNF-α levels in NRF2-proficient BV-2 cells than in NRF2-silenced BV-2 cells. 6-shogaol inhibited 40.35% in NRF2-proficient BV-2 cells and no inhibition in NRF2-silenced BV-2 cells. The most active compound, STC5, inhibited 70.76% of NRF2-proficient BV-2 cells and only 17.04% in NRF2-silenced BV-2 cells.

The effect of 6-shogaol and STCs on COX-2 production is shown in Figure 5K, and the percentage of COX-2 inhibition is shown in Figure 5L. LPS*_Ec_* (200 ng/mL) produced COX-2 in NRF2-proficient BV-2 cells, and the amount was 6.67 ± 0.15 ng/mL. In NRF-2 silenced BV-2 cells, it produced 6.83 ± 0.15 ng/mL. All the STCs and 6-shogaol reversed the elevated levels of COX-2. The compounds STC1 and STC8 are equally active as 6-shogaol. All the other STCs (STC 2-7) are more active than 6-shogaol (4.30 ± 0.10 ng/mL in NRF2-proficient BV-2 cells and 6.03 ± 0.21 ng/mL in NRF2-silenced BV-2 cells). The STC5 is the most active, and COX-2 levels were 27.00 ± 0.10 ng/mL in the NRF2-proficient BV-2 cells and 3.48 ± 0.09 ng/mL in NRF2-silenced BV-2 cells. Among the 2-thiophene analogues, STC8 is the least active; COX-2 levels were 4.10 ± 0.10 ng/mL in the NRF2-proficient BV-2 cells and 4.47 ± 0.15 ng/mL in NRF2-silenced BV-2 cells. The results (Figure 5L) have shown that the 6-shogaol and STCs have more effect on the reversal of elevated COX-2 levels in NRF2-proficient BV-2 cells than in NRF2-silenced BV-2 cells. 6-shogaol inhibited 35.50% in NRF2-proficient BV-2 cells and 11.70% in NRF2-silenced BV-2 cells. The most active compound, STC5, inhibited 76.00% in NRF2-proficient BV-2 cells and only 49.07% in NRF2-silenced BV-2 cells.

The effect of 6-shogaol and STCs on NF-κB p65 production is shown in Figure 5M, and the percentage of COX-2 inhibition is shown in Figure 5N. LPS*_Ec_* (200 ng/mL) produced NF-κB p65 in NRF2-proficient BV-2 cells, and the amount was 125.96 ± 5.04 μg/mL; in NRF-2 silenced BV-2 cells, it produced 138.56 ± 2.52 μg/mL. All the STCs and 6-shogaol reversed the elevated levels of NF-κB p65. The compounds STC1 and STC8 are equally active as 6-shogaol. All the other STCs (STC 2-7) are more active than 6-shogaol (70.54 ± 5.04 ng/mL in NRF2-proficient BV-2 cells and 132.17 ± 4.52 ng/mL in NRF2-silenced BV-2 cells). The STC5 is the most active, and NF-κB p65 levels were 41.48 ± 0.79 μg/mL in the NRF2-proficient BV-2 cells and 86.28 ± 1.03 μg/mL in NRF2-silenced BV-2 cells. Among the 2-thiophene analogues, STC8 is the least active; NF-κB p65 levels were 75.58 ± 5.04 μg/mL in the NRF2-proficient BV-2 cells and 131.57 ± 0.15 ng/mL in NRF2-silenced BV-2 cells. The results (Figure 5N) have shown that the 6-shogaol and STCs have more effect on the reversal of elevated NF-κB p65 levels in NRF2-proficient BV-2 cells than in NRF2-silenced BV-2 cells. 6-shogaol inhibited 44.00% in NRF2-proficient BV-2 cells and 4.61% in NRF2-silenced BV-2 cells. The most active compound, STC5, inhibited 67.07% in NRF2-proficient BV-2 cells and only 37.73%% in NRF2-silenced BV-2 cells.

### 3.5. Mode of Action of STCs on NRF2 Activation

**Figure 6:**
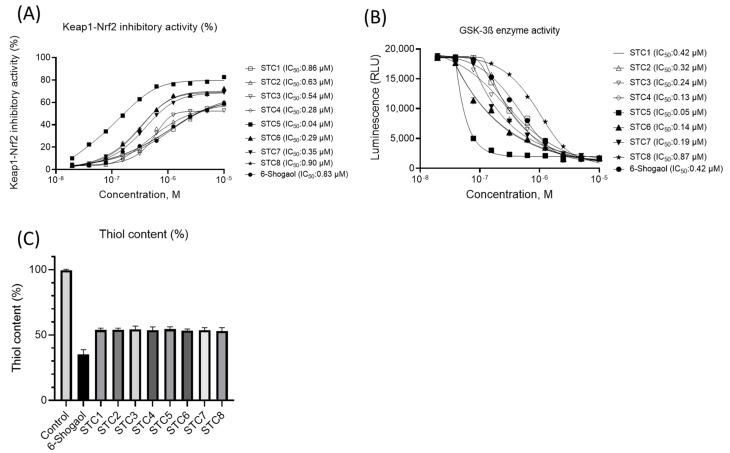
The different modes of NRF2 activation exerted by 6 −shogaol and STCs were determined via (**A**) KEAP1 −NRF2 inhibition, (**B**) GSK-3β enzyme activity, and (**C**) interaction with thiols of cysteine by measuring the free thiol content are shown.

Three different mechanisms mainly activate NRF2 by (1) interacting with the Kelch domain of KEAP1, (2) covalent bonding with cysteine sensors (in BTB domain) of KEAP1, and (3) inhibiting GSK-3β activity. The effect of STCs on the different modes of NRF2 activation is shown in Figure 6. 

The dose-dependent effect of 6-shogaol and STCs on NRF2-KEAP1 inhibition is shown in Figure 6A, and the IC_50_ values (also shown in Figure 6A) are calculated using a four-parametric logistic equation in GraphPad Prism 9.5.0. All the STCs and 6-shogaol inhibited the NRF2-KEAP1 interaction at the KEAP1’s Kelch domain. The IC_50_ value of 6-shogaol is 0.83 ± 0.10 μM. The compounds STC1 (IC_50_, 0.86 ± 0.11 μM) and STC8 (IC_50_, 0.90 ± 0.05 μM) are almost equipotent to 6-shogaol. The most potent compound is STC5, with an IC_50_ value of 0.04 ± 0.001 μM. The potency of STCs in decreasing order is as follows: STC5 > STC4~STC6 > STC7 > STC3 > STC2 > STC1 > STC8. 

The dose-dependent effect of 6-shogaol and STCs on GSK-3β enzyme activity is shown in Figure 6B. The IC_50_ values (Figure 6B) are calculated using a four-parametric logistic equation in GraphPad Prism 9.5.0. All the STCs and 6-shogaol inhibited the GSK-3β enzyme activity. The IC_50_ value of 6-shogaol is 0.42 ± 0.13 μM. The compound STC1 (IC_50_, 0.42 ± 0.08 μM) is equipotent to 6-shogaol. The compound STC8 (IC_50_, 0.87 ± 0.09 μM) is less potent than 6-shogaol. All other STCs (STC2-7) are more potent than 6-shogaol. The most potent compound is STC5, with an IC_50_ value of 0.05 ± 0.001 μM. The potency of STCs in decreasing order is as follows:

STC5 > STC6 > STC4 > STC7 > STC3 > STC2 > STC1 > STC8. 

The 6-shogaol and STCs’ ability to interact with thiols in cysteine residues was indirectly estimated by determining the free thiol content. The free thiol content (%) in NRF2-proficient BV-2 cells upon treatment with test compounds is shown in Figure 6C. The free thiol content in untreated BV-2 cells is assumed as 100%. Treatment of BV-2 cells with 6-shogaol and STCs has shown reduced thiol content, suggesting that all the test compounds interact with thiol groups. The precent free thiol content in 6-shogaol treated BV-2 cells is 35.03 ± 3.70. The free thiol content in STC-treated groups is more than that in 6-shogaol-treated cells, suggesting that the compounds have a lesser (compared to 6-shogaol) ability to interact with cysteine residues. The free thiol content in all the STCs’ treated BV-2 cells is almost similar (~53–54%). 

Overall, the results suggest that STCs have the potential to activate NRF2 through (1) interacting with the Kelch domain of KEAP1, (2) interacting with cysteine residues of KEAP1, and (3) inhibiting GSK-3β enzyme activity. 

### 3.6. Microsomal Stability of STCs 

The metabolic stability of STCs was compared with 6-shogaol in liver microsomes (mouse, rat, and human). The results are shown in Table 2. The microsomal stability of STCs varies in different species. STCs were found to be relatively more stable in liver microsomes than 6-shogaol. The intrinsic clearance values of 6-shogaol were 7.56 ± 1.24 mL/min/g liver, 11.51 ± 1.37 mL/min/g liver, and 14.32 ± 1.51 mL/min/g liver in humans, rats, and mice, respectively. The half-life of 6-shogaol in humans, rats, and mice microsomes are 9.63, 6.32, and 5.08 min, respectively. The intrinsic clearance values of STCs in all three representative liver microsomes were lower than that of 6-shogaol. The half-lives of STCs in all three liver microsomes are greater than 6-shogaol. 

### 3.7. Prediction of Binding Poses and Stability of STCs in the Binding Pockets of Kelch and BTB (Covalent Bonding with Cysteines) Domains of KEAP1 and GSK-3β

**Figure 7:**
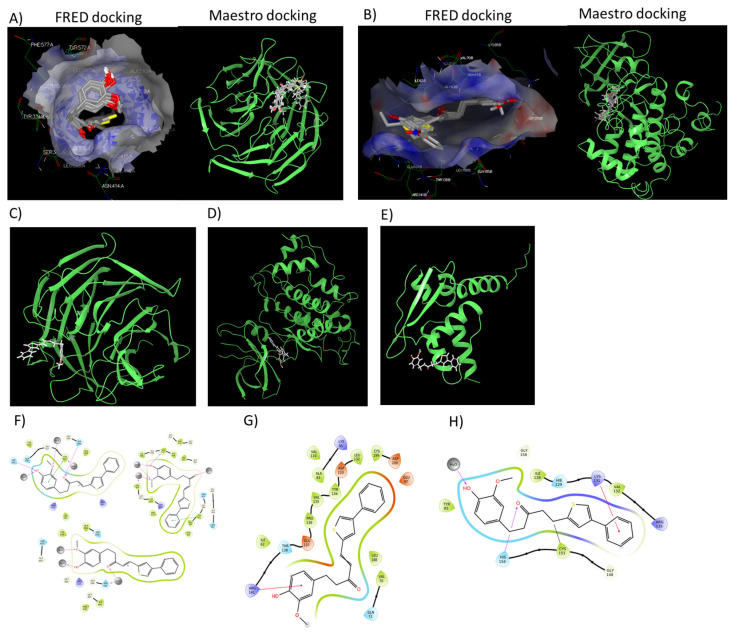
In silico studies of 6-shogaol and STCs with the protein of interest. The in silico studies were performed using FRED and Schrodinger Small Molecule Discovery Suite. The poses of all compounds docked in the (**A**) Kelch protein of KEAP1 (PDB ID: 4IQK) and (**B**) GSK-3β (PDB ID: 1Q41) were visualised using 3-dimensional interaction diagrams. Molecular dynamics studies were carried out on the most potent compound, STC5, with (**C**) Kelch protein of KEAP1 (PDB ID: 4IQK), (**D**) GSK-3β (PDB ID: 1Q41), and (**E**) KEAP1 BTB domain (PDB ID: 5DAF). The 2-dimensional interaction diagram between the STC 5 and the protein of interest, (**F**) 4IQK, (**G**) 1Q41, and (**H**) 5DAF, are shown.

The results of in silico studies are shown in Figure 7 and Table 3. The binding poses of STCs in the binding pockets of KEAP1’s Kelch domain (PDB ID: 4IQK) are shown in Figure 7A, and those in GSK-3β (PDB ID: 1Q41) are shown in Figure 7B. The full details (2D-interaction diagram, drug-like properties, key amino acids involved in binding with the test compounds, and nature of interactions) regarding the binding of STCs are shown in the Supplementary Information. The docking scores (FRED score, XP score) and binding free energies (ΔG^0^) of the test compounds in the binding pockets of 4IQK and 1Q41 are shown in Table 3. The test compounds’ negative docking scores and binding energies in both proteins’ binding sites suggest that their binding is thermodynamically favourable. The stability of the most potent compound, STC5, is further confirmed by MD simulation studies of its complex with 4IQK (shown in Figure 7C), 1Q41 (shown in Figure 7D), and 5DAF (shown in Figure 7E). The MD simulation trajectory of the STC5-4IQK complex suggested three possible binding poses of STC5 in the binding pocket of 4IQK (2D interaction diagrams of three poses of STC5 are shown in Figure 7F). The MD simulation trajectory of the STC5-1Q41 complex suggested only one binding pose of STC5 in the binding pocket of 1Q41 (2D interaction diagram is shown in Figure 7G). The MD simulation trajectory of the STC5-5DAF complex suggested only one binding pose of STC5 in the binding pocket of 5DAF (2D interaction diagram is shown in Figure 7H). The complete MD reports are appended in the Supplementary Information. The results from in silico studies also support the results of lab studies on modes of action of STCs in NRF2 activation as the test compounds form stable complexes with the binding pockets of Kelch domain of KEAP1 (PDB ID: 4IQK), the BTB domain of KEAP1 (PDB ID: 5DAF), and GSK-3β (PDB ID: 1Q41).

## 4. Discussion

Oxidative stress is the leading cause of many chronic diseases. The NRF2 activation is the primary antioxidant defence mechanism to protect from oxidative stress. Thus, NRF2 has emerged as a promising therapeutic target for developing drugs against chronic diseases. Researchers from academia and industry have been extensively investigating to discover new interventions for NRF2 activation. Many NRF2 activating compounds (natural, synthetic, semisynthetic, peptides, etc.) are reported in the literature. Of all these reported compounds, products of natural origin (such as sulforaphane, dimethyl fumarate, monomethyl fumarate, bardoxolone methyl, 6-shogaol, etc.) have shown potent NRF2 activation [16]. Although natural products have shown promising bioactivity (including NRF2 activation), they have shortcomings such as extensive flexibility, poor solubility, poor stability, lower bioavailability, etc. [56]. For example, 6-shogaol is the most potent NRF2 activator at the sub-micromolar range and the most potent bioactive vanilloid, with a wide array of biological activities in chronic diseases. However, 6-shogaol is a very flexible molecule (due to the alkyl side chain) with poor chemical stability, microsomal stability, and aqueous solubility. The α,β-unsaturated carbonyl group in 6-shogaol is reported to be essential for its biological activities, including NRF2 activation. Further, 6-shogaol must be stored at −20 °C to be chemically stable. Therefore, in this study, we have designed the 6-shogaol derivatives by replacing the alkyl side chain with thiophene analogues, with the hypothesis that shogaol thiophene compounds (STCs) possess improved NRF2 activity with improved chemical stability and microsomal stability. The reason for selecting thiophene as a moiety to replace the alkyl group is because (1) organosulfur compounds are reported to activate NRF2 [37,46], and (2) the cysteine (sulfur)-conjugated metabolites of 6-shogaol are more bioactive than 6-shogaol [57]. 

Eight STCs were synthesised (with yields > 85%) using the reusable catalyst proline–proline dipeptide and characterised using NMR (^1^H and ^13^C) and elemental analysis. The polar surface area (predicted values, shown in the Supplementary Information) of all the STCs (except for STC8, which is 89.7 A^2^) is the same as that of 6-shogaol, which is 49.6 A2. The polar surface area is a vital property of the small molecules that determines oral absorption and brain penetration. For passive transportation (by transcellular route) of the drugs, the polar surface area should be < 120 A^2^ for oral absorption and should be < 60A^2^ for brain penetration [58]. Thus, except STC8, all the other STCs can easily penetrate the brain and have the potential to help treat oxidative stress-associated neurogenerative diseases [59], such as Alzheimer’s disease, Huntington’s disease, Parkinson’s disease, etc. Lipophilicity (XlogP is another critical parameter in drug design that determines absorption, distribution, metabolism, excretion, and toxicity (ADMET) properties [60]. According to Lipinski’s rule of 5, the XlogP value should be < 5 for oral absorption. The predicted values of XlogP (shown in the Supplementary Information) for all the STCs is < 5, suggesting these obey Lipinski’s rule of 5 for oral drugs.

The NRF2 activation of the STCs was elucidated by determining the NQO1 induction (Prochaska microtiter plate assay) and expression of representative antioxidant genes (HMOX1, NQO1, GCLC, GCLM, TXNRD1, PRDX1, GPX2, GSTP1, GSTM2, GSTA1, and G6PDX by qPCR) in mouse hepatoma (Hepa-1c1c7) cells. Then, the anti-inflammatory activity of the STCs was investigated in LPS*_Ec_*-challenged mouse microglial (BV-2) cells by determining the levels of inflammation maker (Nitric oxide, NO), proinflammatory cytokines (IL-1β, IL-6, TNF-α, and INF-γ), and inflammatory mediators (COX-2 and NF-κB p65). Nitric oxide production was determined using the Griess assay, and proinflammatory cytokines and inflammatory mediators were determined using ELISA assays. To assess the role of NRF2 activation, the anti-inflammatory activities of the compounds were also assessed in NRF2-silenced BV-2 cells. The modes of action of STCs in NRF2 activation were assessed by measuring their ability to interact with the Kelch domain and cysteine residues of KEAP1 and GSK-3β enzyme activity. The interaction with the Kelch domain and inhibition of GSK-3β enzyme activity was assessed using commercially available kits. The ability of STCs to interact with the cysteine residues was assessed indirectly by determining the free thiol content in NRF2-proficient BV-2 cells upon treatment with STCs. The microsomal stability of the test compounds was assessed in humans, rats, and mice liver microsomes. The binding poses, binding energies, and the key interactions with amino acid residues in the binding pockets of KEAP1’s Kelch domain (4IQK), BTB domain (1Q41), and GSK-3β enzyme were predicted using relevant tools in OpenEye Scientific Software and Schrödinger Drug Discovery Suite. In addition, the stability of the most potent compound (STC5) in the binding pockets of proteins was predicted via molecular dynamics simulation using the Desmond package. In all the experiments, the activities of STCs were compared with those of 6-shogaol. 

In this study, STC1 (containing 3-thiophene) and STC2 (containing 2-thiophene) were initially synthesised and preliminarily evaluated for their NQO1-inducing ability to understand which thiophene gives better activity than 6-shogaol. The results have shown that STC2 is more active than STC1. Thus, six 2-thiophene analogues (STC3-8) containing representative electron-donating (methyl, ethyl), electron-withdrawing (bromo, nitro), and aromatic (phenyl) substituents on 2-thiophene moiety were observed. All the compounds except STC8 (containing nitro substituent) were less cytotoxic to both cell types (Hepa-1c1c7 and BV-2). In the Prochaska microtiter plate assay, STC8 showed toxicity at > 1 μM concentration and did not double the NQO1 induction at concentrations < 1 μM. All other STCs (STC1-7) showed significant NQO1 induction, and STC5 (containing phenyl ring) is the most active compound. The bromo thiophenes (STC4 and STC6) are the second most active compounds, and the bromo substituent’s position does not significantly influence the activity. Among the alkyl-substituted thiophenes, ethylthiophene (STC7) is more active than methylthiophene (STC3). 

All the cell-based biological assays (gene expression and anti-inflammatory studies) were carried out with 1μM effective concentration of STCs. In general, in all the assays, the order of activity follows the NQO1 inducing activity order (STC5 > (STC4~STC6) > STC7 > STC3 > STC 2 > (STC1~6-shogaol) > STC8). The STCs’ order of activity is associated with the order of their XlogP values. STC5 has the highest XlogP of 5.0, and STC8 has the lowest XlogP of 1.8 (the predicted XlogP values are shown in the Supplementary Information). 

NRF2 promotes the transcription of many cytoprotective genes that play a role in oxidative stress-related diseases. Under homeostasis conditions, NRF2 binds with KEAP1 (repressor protein) in the cytoplasm and orchestrates NRF2 ubiquitination. NRF2 activators prevent its ubiquitination and translocate it into the nucleus. Then, NRF2 binds with the antioxidant-response element (ARE) in the nucleus to promote the expression of many antioxidant and detoxification genes, such as HMOX1, NQO1, GCLC, GCLM, TXNRD1, PRDX1, GPX2, GSTP1, GSTM2, GSTA1, and G6PDX [61,62]. Therefore, in this study, we evaluated the ability of the STCs to upregulate the expression of all the abovementioned genes in Hepa-1c1c7 cells. All the STCs have upregulated the expression of antioxidant genes, and the activity order is the same as that observed with NQO1 induction. 

We also show that STCs have the potential to activate NRF2 through three independent mechanisms, i.e., by interacting with the Kelch domain of KEAP1, by interacting with cysteine residues of KEAP1, and by inhibiting GSK-3β enzyme activity. However, we addressed these possibilities primarily using in vitro and in silico approaches, and future experiments are needed to establish which of these mechanisms operate in vivo.

Many reports confirm that NRF2 activation plays a vital role in the anti-inflammatory process in oxidative-stress-associated neurodegenerative diseases [63,64,65]. Therefore, in this study, we evaluated the anti-inflammatory activity of STCs in NRF2-proficient and -silenced BV-2 cells (challenged with LPS*_Ec_*). In both models, the STC2-7 showed significantly improved anti-inflammatory activity compared to 6-shogaol. It is worth noting that the test compounds also showed anti-inflammatory activity in NRF2-silenced BV-2 cells (however, the activity is lower than that observed in NRF2-proficient BV-2 cells), suggesting that other mechanisms might be involved in the anti-inflammatory activity. The compounds have reversed the elevated levels of the inflammatory marker (nitric oxide), cytokines (IL-1β, IL-6, TNF-α, and INF-γ) and mediators (COX-2 and NF-κBP65). 

KEAP1-dependent and KEAP1-independent mechanisms activate NRF2 [66,67], and phytochemicals such as 6-shogaol activate NRF2 through multiple mechanisms [57,68,69]. The KEAP1-dependent activation of NRF2 is mediated via the interaction of NRF2 activators with the Kelch domain and reacting with cysteine residues of KEAP1domains of KEAP1. The KEAP1-independent activation of NRF2 is mediated via the inhibition of GSK-3β enzyme activity. The results from the bioassays have shown that 6-shogaol and STCs activate through three mechanisms of NRF2 activation: (1) binding with the KELCH domain of KEAP1, (2) interacting with cysteine residues of KEAP1, and (3) inhibition of GSK-3β. The stability of the STCs in the binding pockets of the Kelch domain and BTB domain (covalent bonding with cysteine residues) of KEAP1 and GSK-3β was assessed and confirmed using in silico studies (molecular docking and molecular dynamics). Compounds containing α,β-unsaturated carbonyl group (6-shogaol, for example) are reported to be potent NRF2 activators via Michael’s addition reaction with thiols of cysteine residues [33]. However, the main drawback with these compounds is non-selective binding with many proteins, thus leading to side or toxic effects. In this study, we tested the reacting ability of the STCs with free thiols of cysteines in the protein. The results have shown that free thiol content in the cells treated with STCs is higher than that observed in 6-shogaol-treated cells. The reactivity of α,β-unsaturated carbonyls in Michael’s addition reaction depends on the nature of the substituents present in its vicinity [34]. Thus, we postulate that the thiophene’s electron-withdrawing ability and steric hindrance in STCs might be the reason for the lower reactivity of STCs with thiol groups in the cells. 

Metabolic stability refers to the biotransformation of compounds. It plays a vital role in early-phase drug discovery to develop molecules with favourable pharmacokinetic properties [70]. Liver microsomes are employed to determine the in vitro metabolic stability of the compounds. Liver microsomes are subcellular fractions containing drug-metabolising cytochrome P450 enzymes. Liver microsomes are used to determine a compound’s in vitro intrinsic clearance and, thus, metabolic stability. One of the main drawbacks of 6-shogaol is poor metabolic stability with a shorter half-life [71,72]. The α,β-unsaturated carbonyl group in 6-shogaol is responsible for its metabolism: (1) oxidative metabolism by CYP450 enzymes and (2) reductive metabolism by non-CYP450 enzymes. The synthesised 6-shogaol thiophene compounds (STCs) showed improved metabolic stability because they have longer half-lives (T_1/2_) than 6-shogaol in all three representative liver microsomes. The increased metabolic stability of STCs could be because of (1) steric hindrance of α,β-unsaturated carbonyl group by thiophene and (2) reduced electron density on the alkene because of thiophene’s electron-withdrawing ability. 

## 5. Conclusions

In this study, we reported the new 6-shogaol derivatives (shogaol thiophene compounds (STCs)) with improved NRF2 activity through multiple mechanisms (KEAP1-independent and -dependent), decreased reactivity with thiols (that could possibly improve the selectivity in their biological action and reduce the toxicity), and metabolic stability in liver microsomes. The new synthetic method using a reusable catalyst (proline–proline dipeptide) was employed to synthesise STCs in good yields (>85%) and purity (~95%). The drug-like properties and bioactivity of STCs are better than 6-shogaol. The NRF2 activity of STCs significantly depends on the nature of substituents (electron-donating, electron-withdrawing, and aromatic). The STC containing an aromatic ring is the most potent. STCs containing electron-withdrawing show weak activity. STCs containing electron-donating substituents show moderate activity. 6-shogaol activates NRF2 in the micromolar range, whereas the most potent STC (STC5, 65 times more potent than 6-shogaol) activates NRF2 in the sub-micromolar range. The STCs have shown more significant anti-inflammatory effects than 6-shogaol via NRF2-dependent and NRF2-independent mechanisms (future investigations should be carried out to elucidate this aspect further). The half-life of STC5 in liver microsomes is about 1.5 times longer than that of 6-shogaol. The reactivity of STC5 with thiols is about 70% lower than that of 6-shogaol. The compound STC5 has shown promising in vitro activity, whose potential as a drug-like should be further confirmed in animal and pharmacokinetics studies. 

## Figures and Tables

**Scheme 1:**
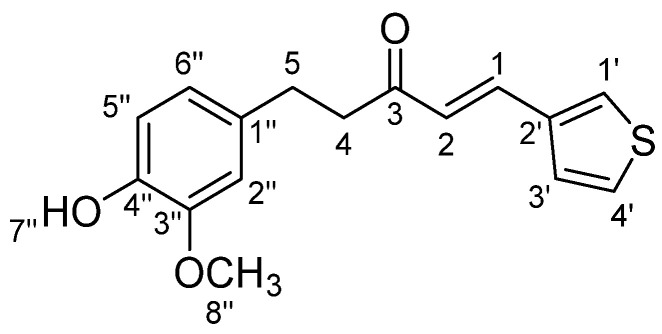
The structure of STC1.

**Scheme 2:**
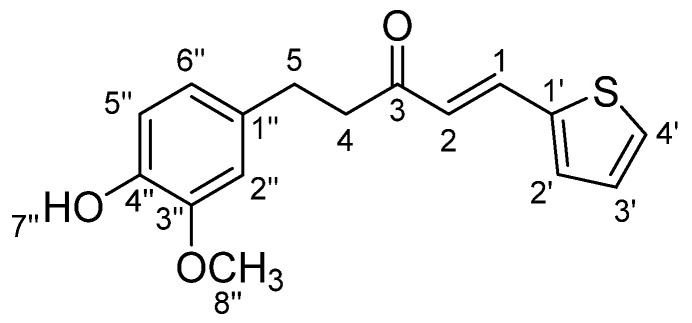
The structure of STC2.

**Scheme 3:**
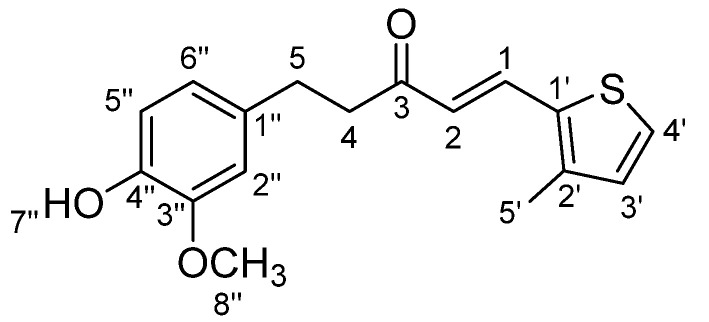
The structure of STC3.

**Scheme 4:**
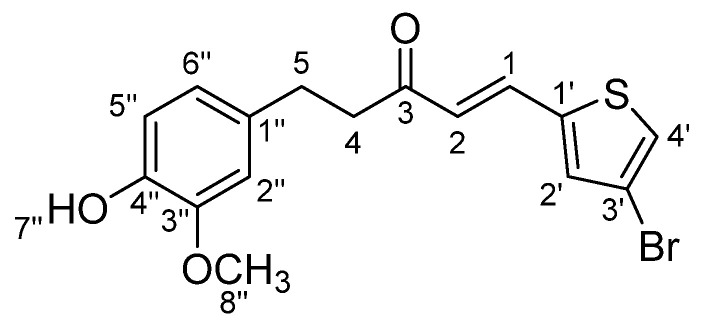
The structure of STC4.

**Scheme 5:**
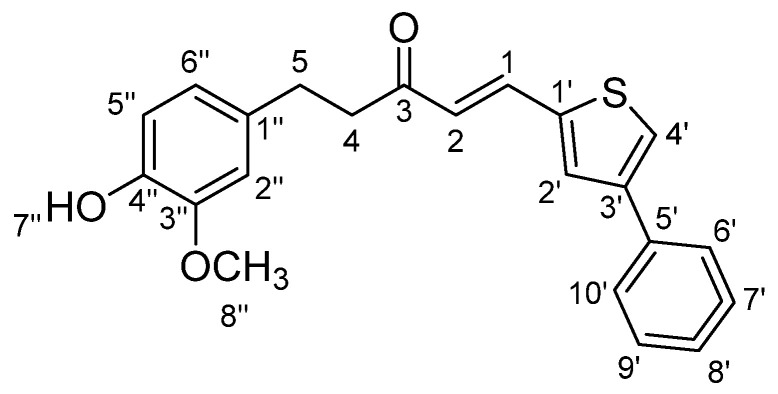
The structure of STC5.

**Scheme 6:**
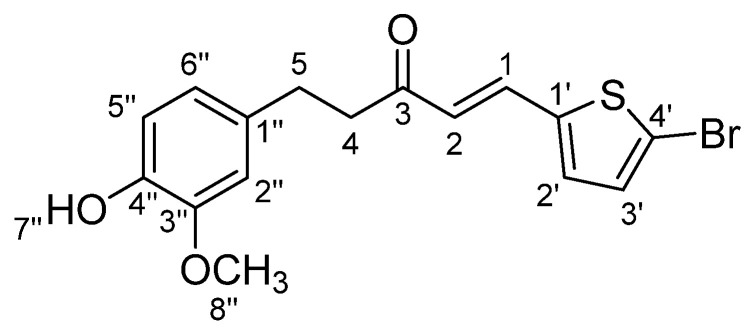
The structure of STC6.

**Scheme 7:**
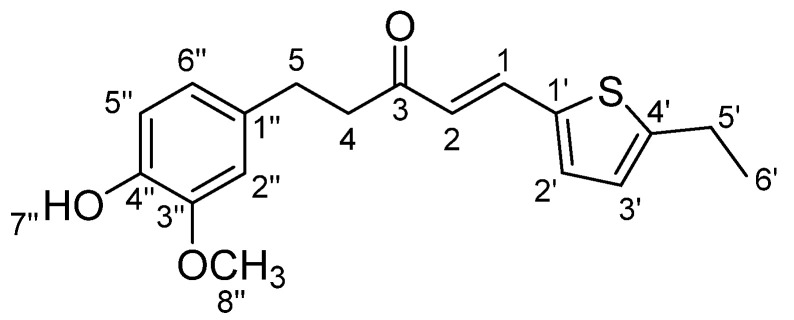
The structure of STC7.

**Scheme 8:**
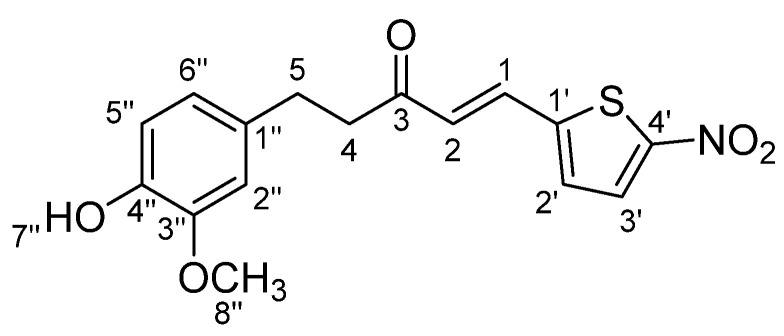
The structure of STC8.

**Table 1:** The genes and primers for qPCR studies.

Gene Symbol	Accession Number	Name	Forward Primer	Reverse Primer
β-Actin	NM_007393.5	Reference gene	CGGTTCCGATGCCCTGAGGCTCTT	CGTCACACTTCATGATGGAATTGA
NRF2	NM_010902.5	Nuclear factor erythroid 2–related factor 2	CAAGACTTGGGCCACTTAAAAGAC	AGTAAGGCTTTCCATCCTCATCAC
NQO1	NM_008706.5	NAD(P)H quinone dehydrogenase 1	AGCTGGAAGCTGCAGACCTG	CCTTTCAGAATGGCTGGCA
HMOX1	NM_010442.2	Heme oxygenase 1	GTGATGGAGCGTCCACAGC	TTGGTGGCCTCCTTCAAGG
GCLC	NM_010295.2	Glutamate-cysteine ligase catalytic subunit	ATCTGCAAAGGCGGCAAC	ACTCCTCTGCAGCTGGCTC
GCLM	NM_008129.4	Glutamate-cysteine ligase modifier subunit	TGGAGCAGCTGTATCAGTGG	AAATCTGGTGGCATCACACA
TXNRD1	NM_015762.2	Thioredoxin reductase 1	AGAAAGTGCTGGTCTTGGATTTTG	ACACGTTCCTCCGAGACCC
PRDX1	NM_011034.5	Peroxiredoxin 1	ACTCAACTGCCAAGTGATTG	TGGGTGTGTTAATCCATGCC
GPX2	NM_030677.2	Glutathione peroxidase 2	TGTCAGAACGAGGAGATCCTG	GACTAAAGGTGGGCTGGTACC
GSTP1	NM_013541.1	Glutathione S-transferase pi 1	GCAAATATGTCACCCTCATCTACACC	GCAGGGTCTCAAAAGGCTTCA
GSTM2	NM_008183.4	Glutathione S-transferase mu 2	GTAGGATTACAAAGCCCAGACCTG	AAGAAATGGAGAGCCCAAGGAC
GSTA4	NM_010357.3	Glutathione S-transferase alpha 4	GGGAACAGTATGAGAAGAAGATGCAAAA	CCCATCGATTTCAACCAAGG
G6PDX	NM_008062.3	Glucose-6-phosphate dehydrogenase X-linked	GTCCAGAATCTCATGGTGCTGA	GCAATGTTGTCTCGATTCCAGA

**Table 2:** The intrinsic clearance and half-life of 6-shogaol and STCs in liver microsomes.

Cmp	Human		Rat		Mouse	
	Cl_int_ (mL/min/g liver)	T_1/2_ (min)	Cl_int_ (mL/min/g liver)	T_1/2_ (min)	Cl_int_ (mL/min/g liver)	T_1/2_ (min)
Ver	11.82 ± 0.07	6.34	14.72 ±0.03	5.29	13.31 ± 0.08	5.74
6-S	7.56 ± 1.24	9.63	11.51 ± 1.37	6.32	14.32 ± 1.51	5.08
STC1	7.15 ± 1.24 *	10.18	10.63 ± 1.25 *	6.85	11.24 ± 1.43 *	6.47
STC2	6.85 ± 1.62 *	10.62	9.46 ± 1.85 *	7.69	10.64 ± 1.66 *	6.84
STC3	6.18 ± 1.34 *	11.77	9.65 ± 1.54 *	7.54	9.27 ± 2.14 *	7.85
STC4	6.35 ± 1.79 *	11.46	8.79 ± 1.94 *	8.28	9.67 ± 1.23 *	7.52
STC5	5.09 ± 0.98 *	14.30	7.22 ± 1.23 *	10.08	6.42 ± 1.52 *	11.33
STC6	5.32 ±1.08 *	13.68	8.17 ± 1.74 *	8.91	7.23 ± 1.34 *	10.06
STC7	6.27 ± 1.44 *	11.61	8.52 ± 1.16 *	8.54	7.85 ± 2.51 *	9.27
STC8	6.95 ± 1.52 *	10.47	10.57 ± 1.48 *	6.88	11.26 ± 2.69 *	6.46

Note: Cl_int_, intrinsic clearance; t_1/2_, half-life; HLM, human liver microsomes; RLM, rat liver microsomes; MLM, mouse liver microsomes; Ver, Verapamil; 6-S, 6-shogaol; Cmp, compound. The data are shown as mean ± SD (n = 9). * Indicates the statistical difference (*p* < 0.05) compared to 6-shogaol.

**Table 3:** The docking scores (FRED score and XP score) and binding free energies (ΔG^0^) of the test compounds in the binding pockets of 4IQK and 1Q41.

PDB ID	Compound	FRED Score	XP Docking (kcal/mole)	Binding Free Energy (ΔG^0^)
4IQK	6−shogaol	−11.92	−5.168	−60.06
	STC1	−11.92	−6.334	−62.71
	STC2	−11.77	−6.237	−61.69
	STC3	−12.06	−5.661	−54.44
	STC4	−12.61	−5.677	−48.83
	STC5	−14.47	−6.819	−62.19
	STC6	−11.96	−5.536	−56.25
	STC7	−12.50	−6.332	−66.91
	STC8	−12.18	−6.111	−46.73
1Q41	6-shogaol	−10.43	−7.725	−42.94
	STC1	−10.82	−8.598	−52.37
	STC2	−10.45	−7.561	−45.27
	STC3	−10.62	−8.561	−50.60
	STC4	−11.18	−8.659	−40.40
	STC5	−11.87	−7.783	−66.23
	STC6	−10.87	−6.557	−49.41
	STC7	−11.15	−7.435	−54.51
	STC8	−11.33	−8.413	−54.17
5DAF	6-shogaol	NA	−3.723	NA
	STC1	NA	−5.498	NA
	STC2	NA	−5.335	NA
	STC3	NA	−5.221	NA
	STC4	NA	−5.804	NA
	STC5	NA	−5.245	NA
	STC6	NA	−5.541	NA
	STC7	NA	−4.736	NA
	STC8	NA	−5.644	NA

Note: PDB ID, protein data bank identification; NA, not available; XP docking, extra precision docking; FRED, fast rigid exhaustive docking.

## Data Availability

Data is contained within the article or supplementary material.

## Supplementary Materials

The supporting information can be downloaded at: https://www.mdpi.com/article/10.3390/antiox12020475/s1. The ^1^H and ^13^C NMR spectra of the compounds are shown in Supplementary Figures S1–S16. NRF2 gene expression in NRF2 siRNA-transfected BV-2 cells is shown in Figure S17. The cytotoxicity of the compounds in Hepa-1c1c7 and BV2 cells are shown in Supplementary Figure S18. The FRED docking reports for all the compounds with two proteins (4IQK and IQ41) are shown in Supplementary Figures S19–S36. The full molecular dynamics reports of the most active compound in three proteins (4IQK, IQ41, and 5DAF) are attached in sequence at the end of the Supplementary Materials.

## References

[1] Dugasani S., Pichika M.R., Nadarajah V.D., Balijepalli M.K., Tandra S., Korlakunta J.N. (2010). Comparative antioxidant and anti-inflammatory effects of [6]-gingerol, [8]-gingerol, [10]-gingerol and [6]-shogaol. J. Ethnopharmacol..

[2] Mai C.-W., Kang Y.B., Hamzah A.S., Pichika M.R. (2018). Comparative efficacy of vanilloids in inhibiting toll-like receptor-4 (tlr-4)/myeloid differentiation factor (md-2) homodimerisation. Food Funct..

[3] Mai C.W., Kang Y.B., Nadarajah V.D., Hamzah A.S., Pichika M.R. (2018). Drug-like dietary vanilloids induce anticancer activity through proliferation inhibition and regulation of bcl-related apoptotic proteins. Phytother. Res..

[4] Ooi S.L., Campbell R., Pak S.C., Golombick T., Manoharan A., Ramakrishna R., Badmaev V., Schloss J. (2021). Is 6-Shogaol an Effective Phytochemical for Patients With Lower-Risk Myelodysplastic Syndrome? A Narrative Review. Integr. Cancer Ther..

[5] Kou X., Wang X., Ji R., Liu L., Qiao Y., Lou Z., Ma C., Li S., Wang H., Ho C.-T. (2018). Occurrence, biological activity and metabolism of 6-shogaol. Food Funct..

[6] Zhang S., Kou X., Zhao H., Mak K.K., Balijepalli M.K., Pichika M.R. (2022). Zingiber Officinale Var. Rubrum: Red Ginger’s Medicinal Uses. Molecules.

[7] Han Q., Yuan Q., Meng X., Huo J., Bao Y., Xie G. (2017). 6-shogaol attenuates lps-induced inflammation in bv2 microglia cells by activating ppar-γ. Oncotarget.

[8] Kode J., Maharana J., Dar A.A., Mukherjee S., Gadewal N., Sigalapalli D.K., Kumar S., Panda D., Ghosh S., Keshry S.S. (2022). 6-shogaol exhibits anti-viral and anti-inflammatory activity in covid-19-associated inflammation by regulating nlrp3 inflammasomes. ACS Omega.

[9] Ajeigbe O.F., Maruf O.R., Anyebe D.A., Opafunso I.T., Ajayi B.O., Farombi E.O. (2022). 6- shogaol suppresses aom/dss-mediated colorectal adenoma through its antioxidant and anti-inflammatory effects in mice. J. Food Biochem..

[10] Patil M.J., Huang Y., Yu M., Dong X., Undem B.J., Yu S. (2022). Ginger constituent 6-shogaol attenuates vincristine-induced activation of mouse gastroesophageal vagal afferent c-fibers. Molecules.

[11] Gratal P., Mediero A., Lamuedra A., Matamoros-Recio A., Herencia C., Herrero-Beaumont G., Martín-Santamaría S., Largo R. (2022). 6-shogaol (enexasogoal) treatment improves experimental knee osteoarthritis exerting a pleiotropic effect over immune innate signalling responses in chondrocytes. Br. J. Pharmacol..

[12] Li N., Li X., Deng L., Yang H., Gong Z., Wang Q., Pan D., Zeng S., Chen J. (2023). 6-shogaol inhibits the proliferation, apoptosis, and migration of rheumatoid arthritis fibroblast-like synoviocytes via the pi3k/akt/nf-κb pathway. Phytomedicine.

[13] Jiao W., Sang Y., Wang X., Wang S. (2023). Metabonomics and the gut microbiome analysis of the effect of 6-shogaol on improving obesity. Food Chem..

[14] Park G., Oh D.S., Lee M.G., Lee C.E., Kim Y. (2016). ung 6-shogaol, an active compound of ginger, alleviates allergic dermatitis-like skin lesions via cytokine inhibition by activating the nrf2 pathway. Toxicol. Appl. Pharmacol..

[15] Chen F., Tang Y., Sun Y., Veeraraghavan V.P., Mohan S.K., Cui C. (2019). 6-shogaol, a active constiuents of ginger prevents uvb radiation mediated inflammation and oxidative stress through modulating nrf2 signaling in human epidermal keratinocytes (hacat cells). J. Photochem. Photobiol. B..

[16] Cuadrado A., Rojo A.I., Wells G., Hayes J.D., Cousin S.P., Rumsey W.L., Attucks O.C., Franklin S., Levonen A.-L., Kensler T.W. (2019). Therapeutic targeting of the nrf2 and keap1 partnership in chronic diseases. Nat. Rev. Drug Discov..

[17] Wang L., Zhang X., Xiong X., Zhu H., Chen R., Zhang S., Chen G., Jian Z. (2022). Nrf2 regulates oxidative stress and its role in cerebral ischemic stroke. Antioxidants.

[18] Tucci P., Lattanzi R., Severini C., Saso L. (2022). Nrf2 pathway in huntington’s disease (hd): What is its role?. Int. J. Mol. Sci..

[19] Dayalan Naidu S., Dinkova-Kostova A.T. (2020). KEAP1, a Cysteine-Based Sensor and a Drug Target for the Prevention and Treatment of Chronic Disease. Open Biol..

[20] Dinkova-Kostova A.T., Abramov A.Y. (2015). The emerging role of nrf2 in mitochondrial function. Free Radic. Biol. Med..

[21] Dinkova-Kostova A.T., Fahey J.W., Kostov R.V., Kensler T.W. (2017). KEAP1 and done? Targeting the nrf2 pathway with sulforaphane. Trends Food Sci. Technol..

[22] Robledinos-Antón N., Fernández-Ginés R., Manda G., Cuadrado A. (2019). Activators and Inhibitors of NRF2: A Review of Their Potential for Clinical Development. Oxid. Med. Cell. Longev..

[23] Zhou Q., Zhang N., Hu T., Xu H., Duan X., Liu B., Chen F., Wang M. (2022). Dietary Phenolic-Type Nrf2-Activators: Implications in the Control of Toxin-Induced Hepatic Disorders. Food Funct..

[24] Iranshahy M., Iranshahi M., Abtahi S.R., Karimi G. (2018). The Role of Nuclear Factor Erythroid 2-Related Factor 2 in Hepatoprotective Activity of Natural Products: A Review. Food Chem. Toxicol..

[25] Jadeja R.N., Upadhyay K.K., Devkar R.V., Khurana S. (2016). Naturally Occurring Nrf2 Activators: Potential in Treatment of Liver Injury. Oxid. Med. Cell. Longev..

[26] Lu M.C., Ji J.A., Jiang Z.Y., You Q.D. (2016). The keap1–nrf2–are pathway as a potential preventive and therapeutic target: An update. Med. Res. Rev..

[27] Sun H., Zhu J., Lin H., Gu K., Feng F. (2017). Recent progress in the development of small molecule nrf2 modulators: A patent review (2012–2016). Expert Opin. Ther. Pat..

[28] Crisman E., Duarte P., Dauden E., Cuadrado A., Rodríguez-Franco M.I., López M.G., León R. (2022). KEAP1-nrf2 protein–protein interaction inhibitors: Design, pharmacological properties and therapeutic potential. Med. Res. Rev..

[29] Zhou H., Wang Y., You Q., Jiang Z. (2020). Recent progress in the development of small molecule nrf2 activators: A patent review (2017-present). Expert Opin. Ther. Pat..

[30] Clifford T., Acton J.P., Cocksedge S.P., Davies K.A.B., Bailey S.J. (2021). The Effect of Dietary Phytochemicals on Nuclear Factor Erythroid 2-Related Factor 2 (Nrf2) Activation: A Systematic Review of Human Intervention Trials. Mol. Biol. Rep..

[31] Zhu Y., Wang P., Zhao Y., Yang C., Clark A., Leung T.C., Chen X., Sang S. (2016). Synthesis, evaluation, and metabolism of novel [6]-shogaol derivatives as potent nrf2 activators. Free Radic. Biol. Med..

[32] Bezerra D.P., Soares A.K.N., De Sousa D.P. (2016). Overview of the Role of Vanillin on Redox Status and Cancer Development. Oxid. Med. Cell. Longev..

[33] Egbujor M.C., Buttari B., Profumo E., Telkoparan-Akillilar P., Saso L. (2022). An overview of nrf2-activating compounds bearing &alpha;,&beta;-unsaturated moiety and their antioxidant effects. Int. J. Mol. Sci..

[34] Jackson P.A., Widen J.C., Harki D.A., Brummond K.M. (2017). Covalent modifiers: A chemical perspective on the reactivity of α,β-unsaturated carbonyls with thiols via hetero-michael addition reactions. J. Med. Chem..

[35] Ahmed S.H.H., Gonda T., Hunyadi A. (2021). Medicinal chemistry inspired by ginger: Exploring the chemical space around 6-gingerol. RSC Adv..

[36] Khanna V., Ranganathan S. (2009). Physiochemical property space distribution among human metabolites, drugs and toxins. BMC Bioinformatics.

[37] Egbujor M.C., Petrosino M., Zuhra K., Saso L. (2022). The role of organosulfur compounds as nrf2 activators and their antioxidant effects. Antioxidants.

[38] Choi J.W., Shin S.J., Kim H.J., Park J.H., Kim H.J., Lee E.H., Pae A.N., Bahn Y.S., Park K.D. (2019). Antioxidant, anti-inflammatory, and neuroprotective effects of novel vinyl sulfonate compounds as nrf2 activator. ACS Med. Chem. Lett..

[39] Zhao C., Rakesh K.P., Ravidar L., Fang W.Y., Qin H.L. (2019). Pharmaceutical and medicinal significance of sulfur (svi)-containing motifs for drug discovery: A critical review. Eur. J. Med. Chem..

[40] Fahey J.W., Dinkova-Kostova A.T., Stephenson K.K., Talalay P. (2004). The “prochaska” microtiter plate bioassay for inducers of nqo1. Methods Enzymol..

[41] Van Muiswinkel F.L., Kuiperij H.B. (2005). The nrf2-are signalling pathway: Promising drug target to combat oxidative stress in neurodegenerative disorders. Curr. Drug Targets CNS Neurol. Disord..

[42] Lim H.-S., Kim Y.J., Kim B.-Y., Park G., Jeong S.-J. (2018). The anti-neuroinflammatory activity of tectorigenin pretreatment via downregulated nf-kappab and erk/jnk pathways in bv-2 microglial and microglia inactivation in mice with lipopolysaccharide. Front. Pharmacol..

[43] Livak K.J., Schmittgen T.D. (2001). Analysis of relative gene expression data using real-time quantitative pcr and the 2(-delta delta c(t)) method. Methods.

[44] Ponthan F., Yusoff N.M., Soria N.M., Heidenreich O., Coffey K. (2015). Gene silencing by rnai in mammalian cells. Curr. Protoc. Mol. Biol..

[45] Michalska P., Mayo P., Fernández-Mendívil C., Tenti G., Duarte P., Buendia I., Ramos M.T., López M.G., Menéndez J.C., León R. (2020). Antioxidant, anti-inflammatory and neuroprotective profiles of novel 1,4-dihydropyridine derivatives for the treatment of alzheimer’s disease. Antioxidants.

[46] Mak K.K., Shiming Z., Epemolu O., Dinkova-Kostova A.T., Wells G., Gazaryan I.G., Sakirolla R., Mohd Z., Pichika M.R. (2022). Synthesis and anti-inflammatory activity of 2-amino-4,5,6,7-tetrahydrobenzo[b]thiophene-derived nrf2 activators. ChemistryOpen.

[47] McGann M. (2012). FRED and hybrid docking performance on standardized datasets. J. Comput. Aided. Mol. Des..

[48] McGann M. (2011). FRED pose prediction and virtual screening accuracy. J. Chem. Inf. Model..

[49] Marcotte D., Zeng W., Hus J.C., McKenzie A., Hession C., Jin P., Bergeron C., Lugovskoy A., Enyedy I., Cuervo H. (2013). Small molecules inhibit the interaction of nrf2 and the keap1 kelch domain through a non-covalent mechanism. Bioorganic Med. Chem..

[50] Bertrand J.A., Thieffine S., Vulpetti A., Cristiani C., Valsasina B., Knapp S., Kalisz H.M., Flocco M. (2003). Structural characterization of the gsk-3β active site using selective and non-selective atp-mimetic inhibitors. J. Mol. Biol..

[51] Kelley B.P., Brown S.P., Warren G.L., Muchmore S.W. (2015). POSIT: Flexible shape-guided docking for pose prediction. J. Chem. Inf. Model..

[52] Hawkins P.C.D., Skillman A.G., Warren G.L., Ellingson B.A., Stahl M.T. (2010). Conformer generation with omega: Algorithm and validation using high quality structures from the protein databank and cambridge structural database. J. Chem. Inf. Model..

[53] Huerta C., Jiang X., Trevino I., Bender C.F., Ferguson D.A., Probst B., Swinger K.K., Stoll V.S., Thomas P.J., Dulubova I. (2016). Characterization of novel small-molecule nrf2 activators: Structural and biochemical validation of stereospecific keap1 binding. Biochim. Biophys. Acta.

[54] Zhu K., Borrelli K.W., Greenwood J.R., Day T., Abel R., Farid R.S., Harder E. (2014). Docking covalent inhibitors: A parameter free approach to pose prediction and scoring. J. Chem. Inf. Model..

[55] Friesner R.A., Murphy R.B., Repasky M.P., Frye L.L., Greenwood J.R., Halgren T.A., Sanschagrin P.C., Mainz D.T. (2006). Extra precision glide: Docking and scoring incorporating a model of hydrophobic enclosure for protein−ligand complexes. J. Med. Chem..

[56] Yao H., Liu J., Xu S., Zhu Z., Xu J. (2016). The structural modification of natural products for novel drug discovery. Expert Opin. Drug Discov..

[57] Chen H., Fu J., Chen H., Hu Y., Soroka D.N., Prigge J.R., Schmidt E.E., Yan F., Major M.B., Chen X. (2014). Ginger compound [6]-shogaol and its cysteine-conjugated metabolite (m2) activate nrf2 in colon epithelial cells in vitro and in vivo. Chem. Res. Toxicol..

[58] Kelder J., Grootenhuis P.D.J., Bayada D.M., Delbressine L.P.C., Ploemen J.P. (1999). Polar molecular surface as a dominating determinant for oral absorption and brain penetration of drugs. Pharm. Res..

[59] Salim S. (2017). Oxidative stress and the central nervous system. J. Pharmacol. Exp. Ther..

[60] Arnott J.A., Planey S.L. (2012). The influence of lipophilicity in drug discovery and design. Expert Opin. Drug Discov..

[61] Tonelli C., Chio I.I.C., Tuveson D.A. (2018). Transcriptional regulation by nrf2. Antioxidants Redox Signal..

[62] Li Y., Paonessa J.D., Zhang Y. (2012). Mechanism of chemical activation of nrf2. PLoS ONE.

[63] Dinkova-Kostova A.T., Kostov R.V., Kazantsev A.G. (2018). The role of nrf2 signaling in counteracting neurodegenerative diseases. FEBS J..

[64] Brandes M.S., Gray N.E. (2020). NRF2 as a Therapeutic Target in Neurodegenerative Diseases. ASN Neuro.

[65] Tu W., Wang H., Li S., Liu Q., Sha H. (2019). The Anti-Inflammatory and Anti-Oxidant Mechanisms of the Keap1/Nrf2/ARE Signaling Pathway in Chronic Diseases. Aging Dis..

[66] Bryan H.K., Olayanju A., Goldring C.E., Park B.K. (2013). The nrf2 cell defence pathway: Keap1-dependent and -independent mechanisms of regulation. Biochem. Pharmacol..

[67] Baird L., Yamamoto M. (2020). The molecular mechanisms regulating the keap1-nrf2 pathway. Mol. Cell. Biol..

[68] Yerer M.B., Tiryaki M.K., Demirpolat E. (2017). GSK-3beta inhibitory effects of 6-gingerol and 6-shogaol help to the recovery of shsy-5y cells after amyloid beta1–42 oligomer or aggregate toxicity. J. Cell. Biotechnol..

[69] Qin S., Hou D.X. (2016). Multiple regulations of keap1/nrf2 system by dietary phytochemicals. Mol. Nutr. Food Res..

[70] Masimirembwa C.M., Bredberg U., Andersson T.B. (2003). Metabolic Stability for Drug Discovery and Development: Pharmacokinetic and Biochemical Challenges. Clin. Pharmacokinet..

[71] Chen H., Soroka D., Zhu Y., Sang S. (2013). Metabolism of ginger component [6]-shogaol in liver microsomes from mouse, rat, dog, monkey, and human. Mol. Nutr. Food Res..

[72] Mukkavilli R., Yang C., Tanwar R.S., Ghareeb A., Luthra L., Aneja R. (2017). Absorption, metabolic stability, & pharmacokinetics of ginger phytochemicals. Molecules.

